# Anticancer effects of the combined Thai noni juice ethanolic extracts and 5-fluorouracil against cholangiocarcinoma cells in vitro and in vivo

**DOI:** 10.1038/s41598-021-94049-z

**Published:** 2021-07-21

**Authors:** Jeerati Prompipak, Thanaset Senawong, Banchob Sripa, Albert J. Ketterman, Suppawit Utaiwat, Khanutsanan Woranam, Jarckrit Jeeunngoi, Gulsiri Senawong

**Affiliations:** 1 Department of Biochemistry, Faculty of Science, Khon Kaen University, Khon Kaen, 40002 Thailand; 2 Natural Product Research Unit, Faculty of Science, Khon Kaen University, Khon Kaen, 40002 Thailand; 3 Department of Pathology, Faculty of Medicine, Khon Kaen University, Khon Kaen, 40002 Thailand; 4 Institute of Molecular Biosciences, Mahidol University, Salaya Campus, Nakhon Pathom, 73170 Thailand

**Keywords:** Biochemistry, Drug discovery, Oncology

## Abstract

Application of 5-fluorouracil (5-FU) in cholangiocarcinoma (CCA) is limited by adverse side effects and chemoresistance. Therefore, the combination therapy of 5-FU with other substances, especially natural products may provide a new strategy for CCA treatment. The aim of this study was to evaluate the combination effects of 5-FU and two ethanolic extracts of Thai noni juice (TNJ) products on CCA cell lines and nude mice xenografts. The results of antiproliferative assay showed the combination treatment of 5-FU and each TNJ ethanolic extract exerted more cytotoxicity on CCA cells than either single agent treatment. Synergistic effects of drug combinations can enable the dose reduction of 5-FU. The mechanism underlying a combination treatment was apoptosis induction through an activation of p53 and Bax proteins. In the nude mouse xenograft model, combination treatments of 5-FU with each TNJ ethanolic extract suppressed the growth of CCA cells implanted mice more than single agent treatments with no effects on mouse body weight, kidney, and spleen. Moreover, low doses of TNJ ethanolic extracts reduced the hepatotoxicity of 5-FU in nude mice. Taken together, these data suggested that the ethanolic extracts of TNJ products can enhance the anti-CCA effect and reduce toxicity of 5-FU.

## Introduction

Cholangiocarcinoma (CCA) is a malignancy that initiates from the biliary epithelium. Depending on the anatomical obstruction and location, CCA is classically subdivided into intrahepatic CCA (10%), perihilar CCA (50–60%) and distal CCA (20–30%)^1^. Global incidence and mortality rates of CCA have been reported and the highest incidence rate of CCA has been documented in northeastern Thailand with 85 cases per 100,000 population^2^. *Opisthorchis viverrine* infection is a main risk factor of CCA in Thailand and other risk factors contribute to CCA development, including bile duct cysts, Caroli's disease, primary sclerosing cholangitis, hepatolithiasis, cholelithiasis, cirrhosis and chronic viral hepatitis^1^. Surgical resection is the only curative treatment that suggestively improves the survival of CCA patients at an early stage. However, CCA in northeast Thailand has a very poor prognosis and most patients have been diagnosed at an advanced stage of the disease in which surgical resection is not possible^3,4^. Therefore, chemotherapy is an important strategy for CCA treatment.

Several chemotherapeutic drugs have been used for CCA treatment such as 5-fluorouracil (5-FU), cisplatin (CIS), and gemcitabine (GEM)^5^. 5-FU, pyrimidine analog, is a chemotherapy regimen for CCA treatment that affects DNA synthesis by blocking the activity of thymidylate synthase leading to DNA strand break^6^. As with most chemotherapeutic drugs, 5-FU resistance is generally an event of altered drug metabolism followed by tumor regrowth and increasing drug dosage. The development of 5-FU resistance in CCA is associated with the up-regulation of thymidylate synthase^7^. In clinical situations, administration of 5-FU alone produced a low response rate, but the combination of 5-FU with other anticancer drugs improved the response rate in biliary tract cancers^8^. Moreover, clinical application of 5-FU causes several side effects such as hepatotoxicity, decreased immunity, diarrhea, nausea, and sensory neuropathy^9^. However, 5-FU remains widely used because of its low cost. Thus, the development of less toxic and more effective anticancer agents in combination with 5-FU is urgently needed. Accordingly, natural products are an interesting source of several phytochemicals with potential to be chemotherapeutic agents. Nowadays, the combination therapy of chemo-natural products is considered as promising.

*Morinda citrifolia* L., commonly known as noni and called Yor in Thailand, is a traditional plant in tropical Asia that is widely used as a functional food and medicinal herb. Noni has been reported to have many pharmacological properties, such as antimicrobial, antifungal, antioxidant, anti-arthritic, anti-inflammatory and anticancer activity^10^. Currently, noni juice has been used as an herbal drink and is commercially available worldwide. Noni fruit juice is a natural source of numerous bioactive compounds, including phenolic acids, flavonoids, iridoids and coumarin^11^. Previous research revealed that the noni juice does not induce adverse effects in hepatocytes, and renal function or show subchronic oral toxicity^12,13^. Moreover, several reports suggest that noni juice has anticancer properties and combination treatment with noni juice can improve response rate and reduce the toxicity of current chemotherapy drugs^14–16^. However, the effects of noni juice in combination treatment with chemotherapeutic agents on CCA have not been reported.

To date, Thai noni juice (TNJ) products are distributed in the local market and worldwide. Several brands of TNJ product are produced from different regions which have been approved by the Food and Drug Administration of Thailand. Although the commercial TNJ has been claimed to have several health benefits, detailed studies on the anticancer effects in combination treatment with chemotherapy drugs have not been reported. In our previous study, two commercial ethanolic extracts of TNJ products, Thai noni juice Chiangrai (TNJ-Cr) and Thai noni juice Buasri (TNJ-Bs), induced apoptosis in two CCA cell lines, KKU-100 and KKU-213B, by up-regulating the expression of Bax through a p53-dependent mechanism^17^. The partial phenolic profiles of both TNJ ethanolic extracts were evaluated by HPLC and the identified phenolic acids included gallic, protocatechuic, *p*-hydroxybenzoic, vanillic, syringic, *p*-coumaric, and ferulic acids. Some major phenolic acids including *p*-hydroxybenzoic, vanillic, and protocatechuic acids were identified in both TNJ ethanolic extracts, however, the highest HPLC peaks of both extracts remain to be identified. In this study, the anti-CCA effects of single and combination agent treatments between 5-FU and two TNJ ethanolic extracts (TNJ-Cr or TNJ-Bs) were evaluated on CCA cell lines and on nude mice xenografts. The mechanisms underlying their synergism were also determined. This finding may provide a new strategy for CCA treatment.

## Results

### Antiproliferative effects of the single agent treatment for 5-FU or TNJ ethanolic extracts

**Figure 1:**
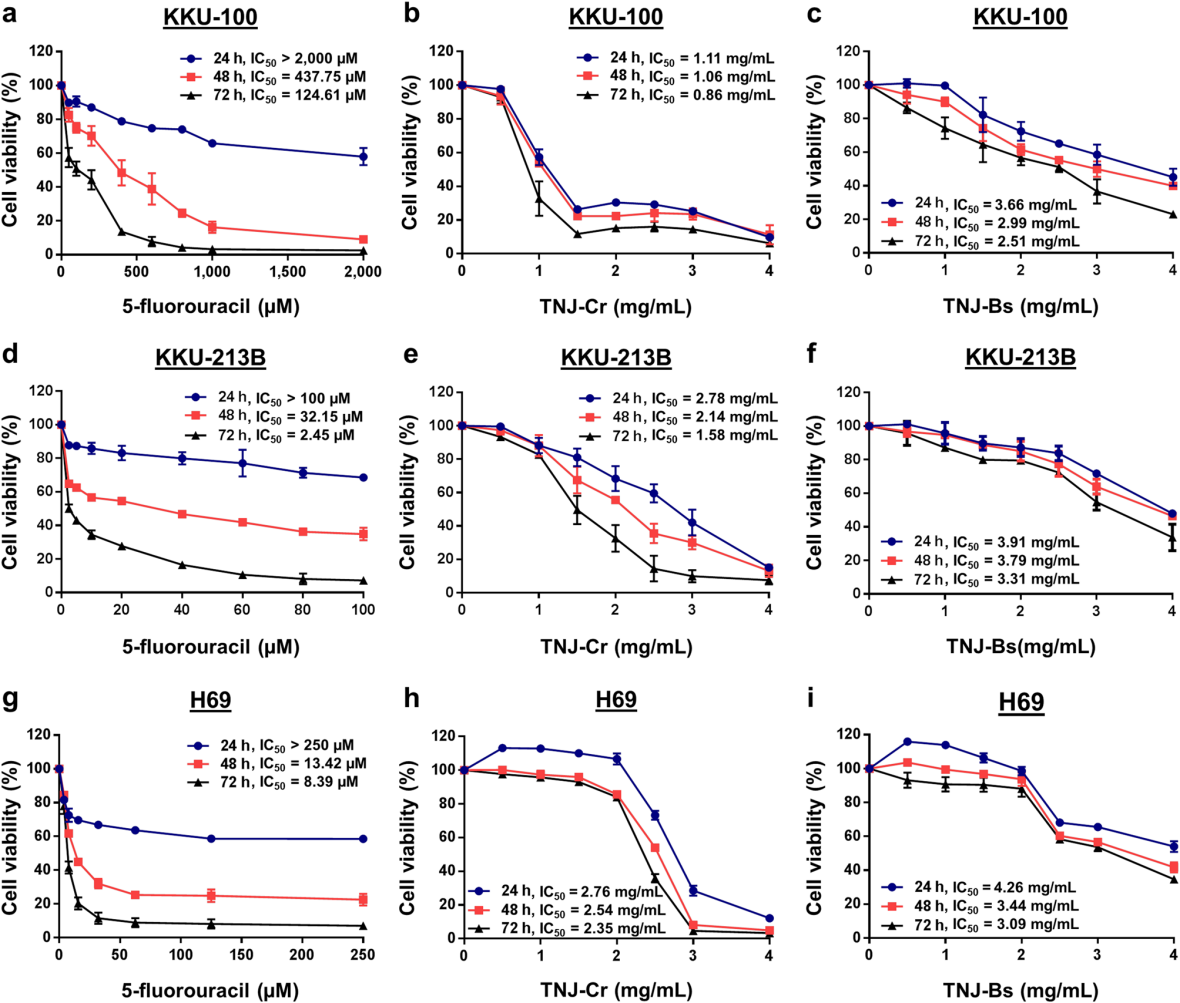
Antiproliferative effects of the single agent treatment among 5-FU and TNJ ethanolic extracts. KKU-100 (**a**–**c**), KKU-213B (**d**–**f**) and H69 (**g**–**i**) cells were treated with various concentrations of 5-FU or TNJ ethanolic extracts for 24, 48 and 72 h. The percentages of cell viability were calculated relative to the solvent control (0.50% ethanol + 0.50% DMSO).

The cytotoxic effects of 5-FU and TNJ ethanolic extracts were determined by using the 3-(4, 5-dimethylthiazol-2-yl)‑2,5-diphenyltetrazolium bromide (MTT) assay. The results presented in Fig. 1 show the percentage of cell viability was dose- and time-dependent. The half-maximal inhibitory concentration (IC_50_) values of each single agent were essentially used to evaluate cellular sensitivity and determine the combination treatment. 5-FU significantly suppressed cell growth of KKU-100 and KKU-213B (Fig. 1a,d) with IC_50_ values of 437.75 ± 99.82 µM (56.94 ± 0.05 µg/mL) and 32.15 ± 2.02 µM (4.18 ± 0.01 µg/mL) for 48 h, respectively. While the ethanolic extract of TNJ-Cr reduced viability of KKU-100 and KKU-213B cells with IC_50_ values of 1.06 ± 0.03 mg/mL and 2.14 ± 0.08 mg/mL for 48 h, respectively (Fig. 1b,e). The ethanolic extract of TNJ-Bs inhibited cell proliferation of both KKU-100 and KKU-213B (Fig. 1c,f) with IC_50_ values of 2.99 ± 0.22 mg/mL and 3.79 ± 0.09 mg/mL, respectively. Furthermore, the IC_50_ of 5-FU on non-cancer cell line H69 for 48 h was 13.42 ± 0.56 µM (1.74 ± 0.07 µg/mL) (Fig. 1g), while the IC_50_ of TNJ-Cr and TNJ-Bs ethanolic extracts on H69 cells at 48 h exposure were 2.49 ± 0.02 mg/mL and 3.40 ± 0.06 mg/mL, respectively (Fig. 1h,i).

### Antiproliferative effects of the combination treatment of 5-FU and TNJ ethanolic extracts

**Figure 2:**
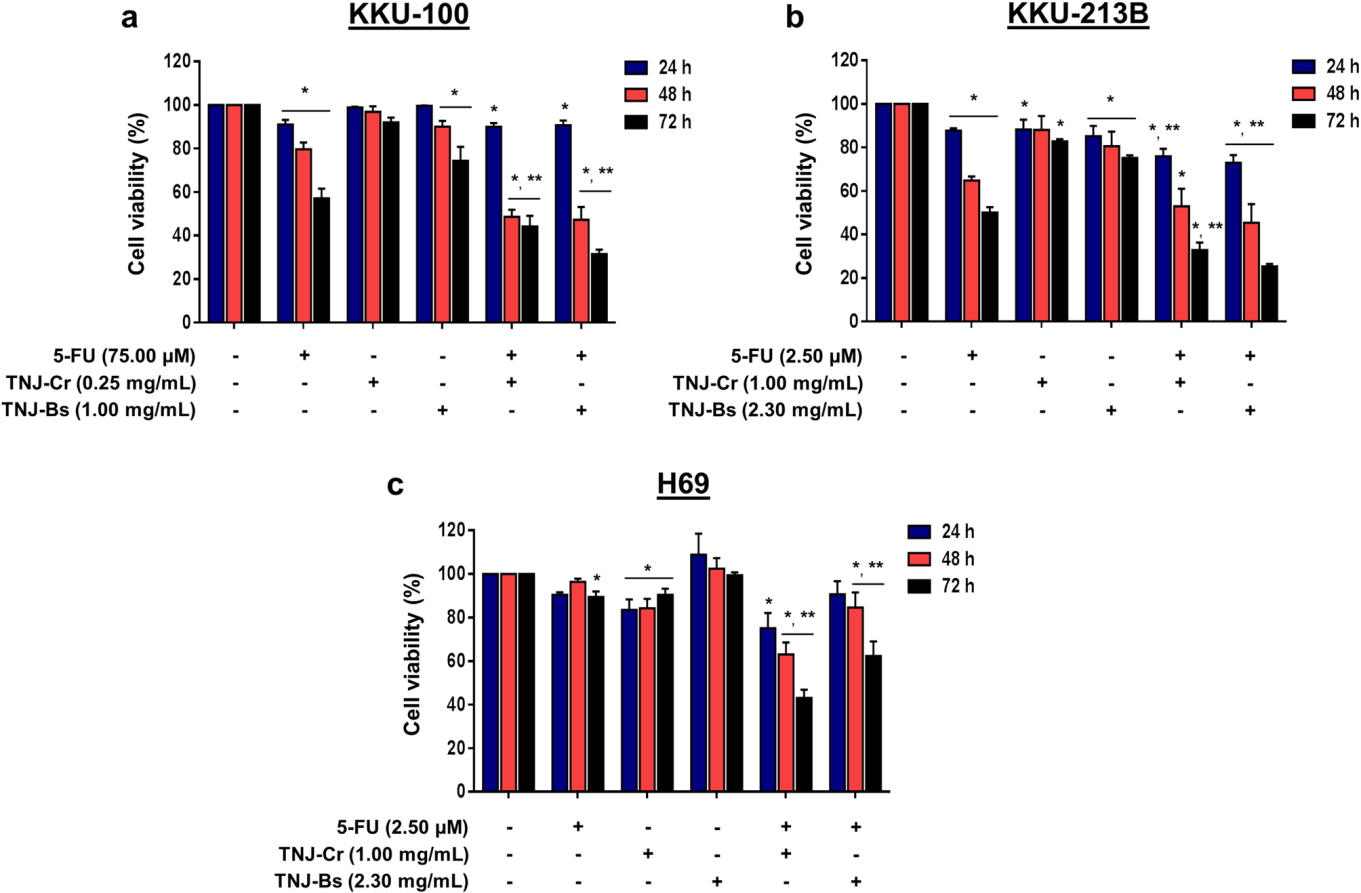
Antiproliferative effects of the combination treatment between 5-FU and TNJ ethanolic extract. CCA cells KKU-100 (**a**), KKU-213B (**b**) and non-cancer cells H69 (**c**) were treated with various concentrations of 5-FU and TNJ ethanolic extracts alone or in combination for 24, 48 and 72 h. The percentages of cell viability were calculated by comparing with the solvent control (0.50% ethanol + 0.50% DMSO). The data are represented as mean ± SD of three independent experiments. *p < 0.05 indicates a significant difference between the treatments and the solvent control; **p < 0.05 indicates a significant difference between the single and combined agent treatments.

The combined effects of 5-FU with the various concentrations of each TNJ ethanolic extract on CCA and non-cancer cells were determined. The results of synergistic effects between combined treatments of 5-FU and TNJ-Cr or TNJ-Bs ethanolic extracts on KKU-100, KKU-213B and H69 cells are shown in Fig. 2. Combination treatments of 5-FU and TNJ ethanolic extracts at 48 h significantly inhibited CCA cell proliferation more than either single agent treatment at the synergistic concentrations for 48 h. As shown in Fig. 2a, cell viability of KKU-100 significantly decreased to 48.55 ± 3.29% or 47.26 ± 5.81% in the combined treatments of 5-FU and TNJ-Cr or TNJ-Bs ethanolic extracts for 48 h, respectively. While 5-FU alone reduced KKU-100 viability to 79.66 ± 3.05% at 48 h of treatment. The ethanolic extracts of TNJ-Cr or TNJ-Bs alone reduced KKU-100 viability at 48 h incubation to 94.04 ± 1.68% or 89.78 ± 3.94%, respectively. Likewise, 5-FU combined with TNJ-Cr or TNJ-Bs ethanolic extracts significantly reduced cell viability of KKU-213B at 48 h to 52.91 ± 8.11% or 45.43 ± 8.56%, respectively, that was more than either single agent treatment at the synergistic concentrations for 48 h (64.18 ± 1.78% for 5-FU, 88.10 ± 6.32% for TNJ-Cr ethanolic extract and 80.39 ± 6.70% for TNJ-Bs ethanolic extract) (Fig. 2b). To elucidate the toxicity of the combined treatments on non-cancer H69 cell, the highest concentration of the combined agents of 5-FU with TNJ-Cr or TNJ-Bs ethanolic extracts that resulted in synergism in CCA cells were used to treat H69. In Fig. 2c, 5-FU and ethanolic extract of TNJ-Cr alone reduced cell viability of H69 at 48 h to 96.41 ± 1.40% and 84.31 ± 4.32%, respectively. The ethanolic extracts of TNJ-Bs alone, did not decrease cell viability of H69 (102.47 ± 4.81%) at 48 h. Whereas the combined treatments between 5-FU and TNJ-Cr or TNJ-Bs ethanolic extracts reduced H69 cell viability at 48 h to 63.10 ± 5.46% and 84.62 ± 6.90%, respectively. Although, the non-cancer H69 cells showed less sensitivity to the combined treatments of 5-FU with TNJ ethanolic extracts than the CCA cells, the combined treatment of 5-FU and TNJ-Cr ethanolic extract at 48 h was relatively toxic to H69 cells.

### Combination index and dose reduction index of the combination treatments of 5-FU and TNJ ethanolic extracts

To determine the combined effects of 5-FU with the ethanolic extract of TNJ-Cr or TNJ-Bs, the combination index (CI) and dose reduction index (DRI) values were calculated to determine the type of drug interaction following the Chou-Talalay method. The sub-toxic dose of 5-FU (the dose causing growth inhibition approximately 20%) was fixed for each CCA cell line in the combination treatments with the various concentrations of each TNJ ethanolic extract (Supplementary Fig. S1). The results revealed the combination treatment of 5-FU with ethanolic extracts of TNJ-Cr or TNJ-Bs at 48 h exposure gave CI values less than 1 which indicates a synergistic effect in both KKU-100 and KKU-213B cells (Table 1). In addition, these synergistic effects at 48 h suggested a dose reduction of 5.83- to 12.86-fold for 5-FU and 1.66- to 4.44-fold for the TNJ ethanolic extracts.

**Table 1:** CI and DRI of the combination treatments of 5-FU and TNJ ethanolic extracts on CCA cells.

Cell lines	Drug combination	Exposure time (h)	CI	DRI		
				5-FU	TNJ-Cr	TNJ-Bs
KKU-100	5-FU + TNJ-Cr	48	0.43	5.83	4.44	–
		72	0.47	4.98	4.53	–
	5-FU + TNJ-Bs	48	0.52	5.83	–	3.37
		72	0.75	4.98	–	2.19
KKU-213B	5-FU + TNJ-Cr	48	0.64	12.86	1.96	–
		72	1.80	2.46	1.01	–
	5-FU + TNJ-Bs	48	0.72	12.86	–	1.66
		72	1.22	2.46	–	1.73

CI: combination index; DRI: dose reduction index; TNJ: Thai noni juice; CCA: cholangiocarcinoma; 5-FU: 5-fluorouracil; TNJ-Cr: ethanolic extract of Thai noni juice Cr; TNJ-Bs: ethanolic extract of Thai noni juice Bs.

### Effect of the combination treatment of 5-FU and TNJ ethanolic extracts on cell cycle distribution and apoptosis induction

**Figure 3:**
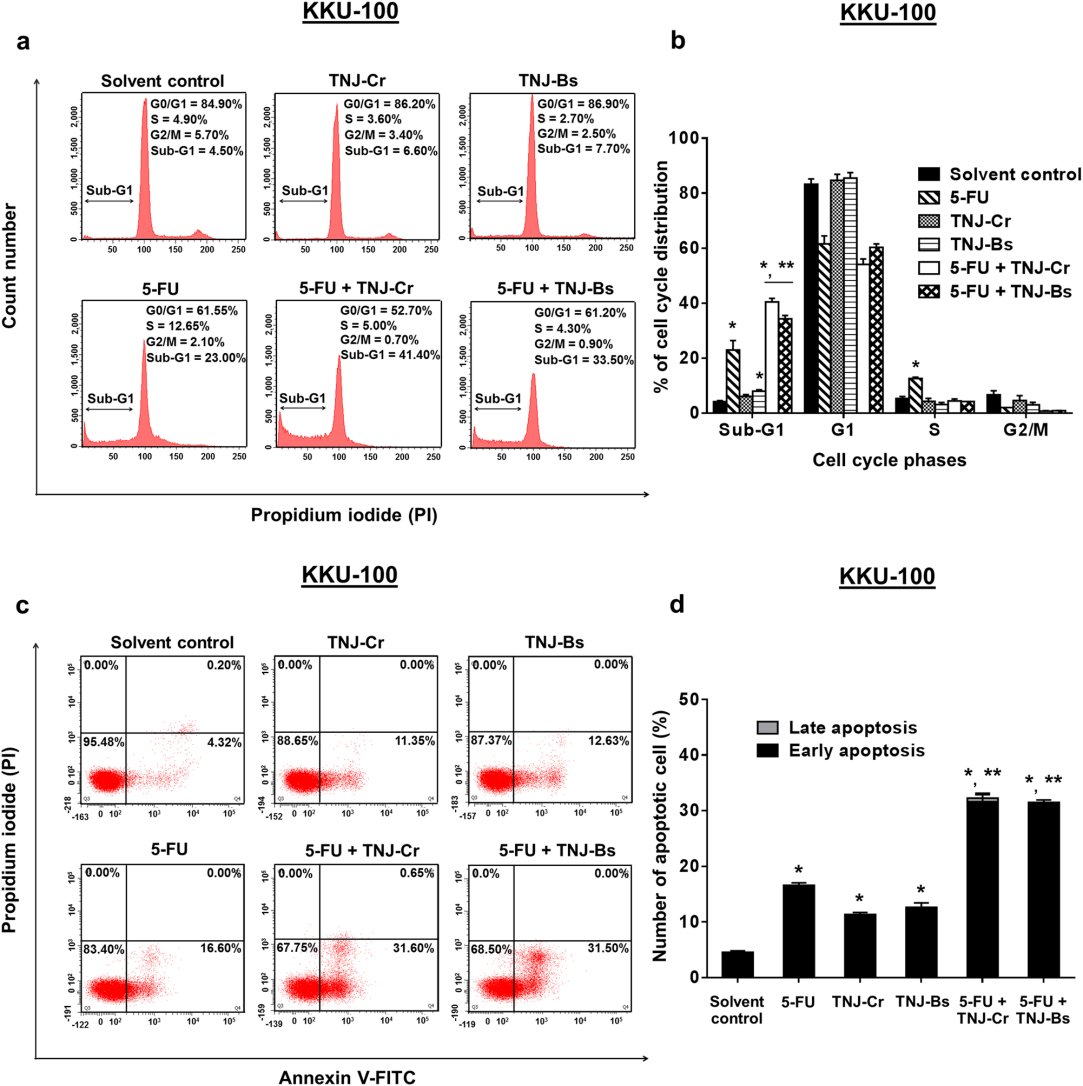
Effects of 5-FU, ethanolic extracts of TNJ-Cr and TNJ-Bs in single or combined agent treatments on cell cycle distribution and apoptosis induction. DNA histograms represent the cell cycle profile of KKU-100 cells after treatments (**a**). The percentage of cell cycle phases are shown as bar graphs of the mean from three independent experiments (**b**). The dot plot represents the flow analysis of apoptosis induction in KKU-100 cells (**c**). The bar graph shows the mean of the percentage of apoptotic cells from three independent experiments (**d**). KKU-100 were treated with solvent (0.25% ethanol + 0.25% DMSO), 5-FU (75 µM), TNJ-Cr (0.25 mg/mL), TNJ-Bs (1.00 mg/mL), 5-FU (75 µM) + TNJ-Cr (0.25 mg/mL) and 5-FU (75 µM) + TNJ-Bs (1.00 mg/mL) for 48 h exposure. *p < 0.05 indicates a significant difference between the treatments and the solvent control; **p < 0.05 indicates a significant difference between the single and combined agent treatments.

To investigate the cell cycle arrest and apoptosis induction in the combination treatment of 5-FU and TNJ ethanolic extracts, the drug resistant KKU-100 cells were chosen as they showed the strongest synergism for the combination treatments. The dose and exposure time of combined drugs that caused the synergism with 50% inhibition of cell proliferation was used to conduct the cell cycle analysis and apoptosis assay in KKU-100 cell. As shown in Fig. 3a,b, the treatment of 5-FU alone at IC_20_ (75 µM) increased the populations in sub-G1 23 ± 3.39% and in S phase 12.65 ± 0.49%, while the ethanolic extracts of TNJ-Cr or TNJ-Bs alone at the synergistic concentration did not affect any cell cycle phase in KKU-100, but slightly increased the sub-G1 population (6.15 ± 0.63% for 0.25 mg/mL TNJ-Cr and 8.05 ± 0.49% for 1.00 mg/mL TNJ-Bs). In the combination treatment, 5-FU combined with TNJ ethanolic extracts did not cause cell cycle arrest but instead greatly increased the sub-G1 population to 40.45 ± 1.34% for 5-FU combined with TNJ-Cr ethanolic extract and 34.35 ± 1.20% for 5-FU combined with TNJ-Bs ethanolic extract, respectively. These results indicated apoptosis was involved in the combination treatment, but not cell cycle arrest. Subsequently, apoptotic induction in KKU-100 cells was determined by annexin V-FITC and PI staining. The results showed that combination treatments of 5-FU (75 µM) and ethanolic extracts of TNJ-Cr (0.25 mg/mL) or TNJ-Bs (1.00 mg/mL) induced apoptosis in KKU-100 cells 32.25 ± 0.35% and 31.50 ± 0.42%, respectively (Fig. 3c,d). By contrast, 5-FU alone induced apoptosis in KKU-100 only 16.60 ± 0.42%, while ethanolic extracts of TNJ-Cr or TNJ-Bs alone induced KKU-100 apoptosis 11.35 ± 0.35% or 12.65 ± 0.77%, respectively. More than the single agent treatments, the combination treatments of 5-FU and TNJ ethanolic extracts significantly increased apoptosis of the drug resistant KKU-100 cells.

### Effect of 5-FU combined with TNJ ethanolic extracts on apoptosis-related proteins and ERK signaling

**Figure 4:**
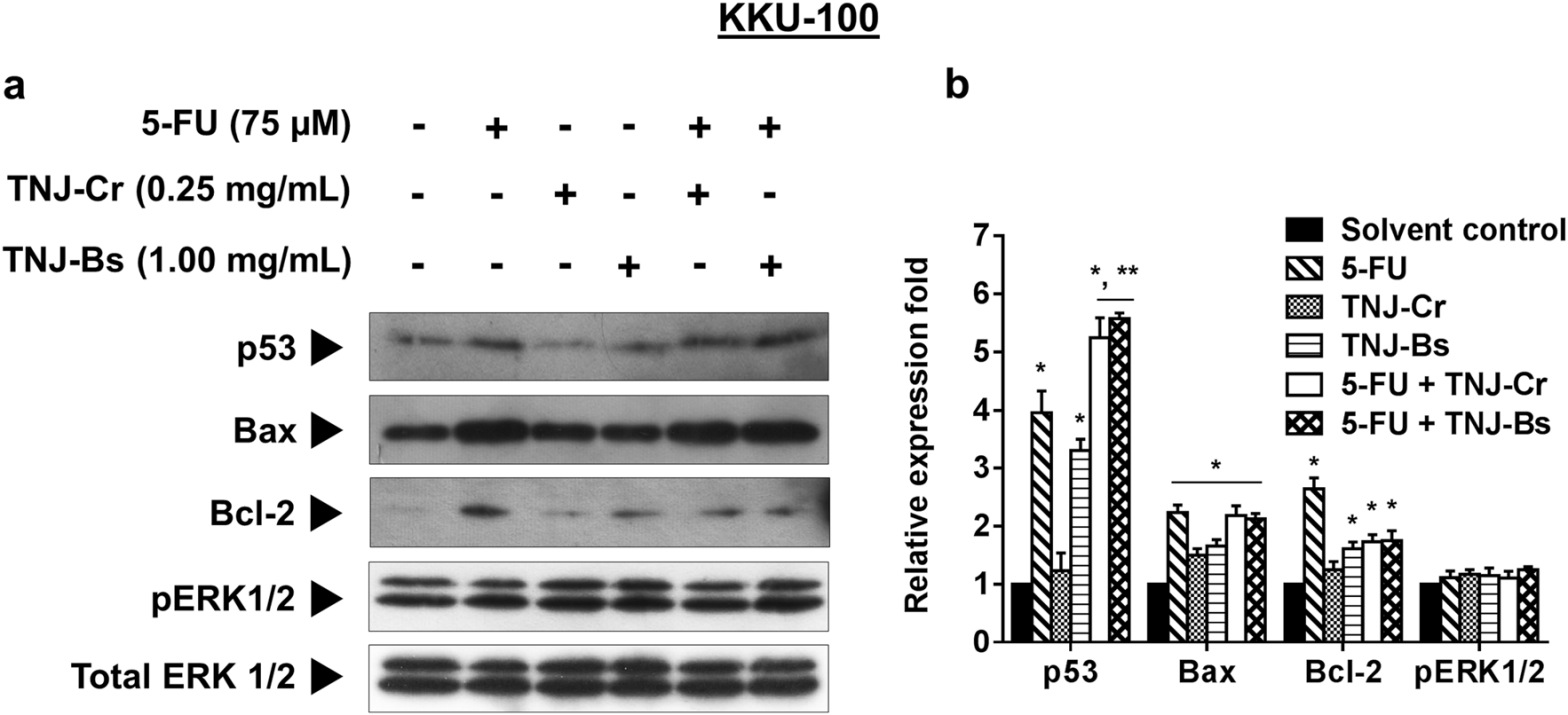
Western blot analysis of apoptosis-related proteins and ERK signaling. Cells were treated with solvent control (0.25% ethanol + 0.25% DMSO), 5-FU (75 µM), TNJ-Cr (0.25 mg/mL), TNJ-Bs (1.00 mg/mL), 5-FU (75 µM) + TNJ-Cr (0.25 mg/mL) and 5-FU (75 µM) + TNJ-Bs (1.00 mg/mL) for 48 h. Total ERK1/2 was used as a loading control (**a**). The relative fold of protein expression was shown as a bar graph from the intensity of the protein band compared with a loading control. Bar graph represents mean ± SD from three independent experiments (**b**). *p < 0.05 indicates a significant difference between the treatments and the solvent control; **p < 0.05 indicates a significant difference between the single and combined agent treatments. The blot images are shown in Supplementary Fig. S3.

Our data revealed that the combination treatment of 5-FU and TNJ ethanolic extracts induces apoptosis in drug resistant KKU-100 cells. To assess the factors involved with apoptosis response at the protein level, western blot analysis was used to evaluate the expression levels of pro-apoptotic (p53, Bax), anti-apoptotic (Bcl-2) and ERK signaling (pERK 1/2). As depicted in Fig. 4, the treatment of 5-FU or ethanolic extract of TNJ-Bs alone up-regulated p53 in KKU-100. Moreover, the relative expression of p53 was significantly increased in the combination treatment of 5-FU and the TNJ ethanolic extracts. The Bax expression level was also increased in KKU-100 cells with single or combined treatment of 5-FU and TNJ ethanolic extracts compared to the solvent control. Although the level of anti-apoptotic protein Bcl-2 was increased in both single and combined agent treatments of KKU-100, the combination treatment between 5-FU and TNJ ethanolic extracts increased the ratio of Bax/Bcl-2 (Supplementary Fig. S2) when compared to the 5-FU treatment alone. However, the expression level of pERK1/2 in KKU-100 cells was not affected by any single or combined agent treatments. The western blot results indicate apoptosis induction in KKU-100 cells upon the combination treatment of 5-FU and TNJ ethanolic extracts may be via p53 and Bax pathways.

### Antitumor effect of 5-FU and TNJ ethanolic extracts on CCA xenograft mice

**Figure 5:**
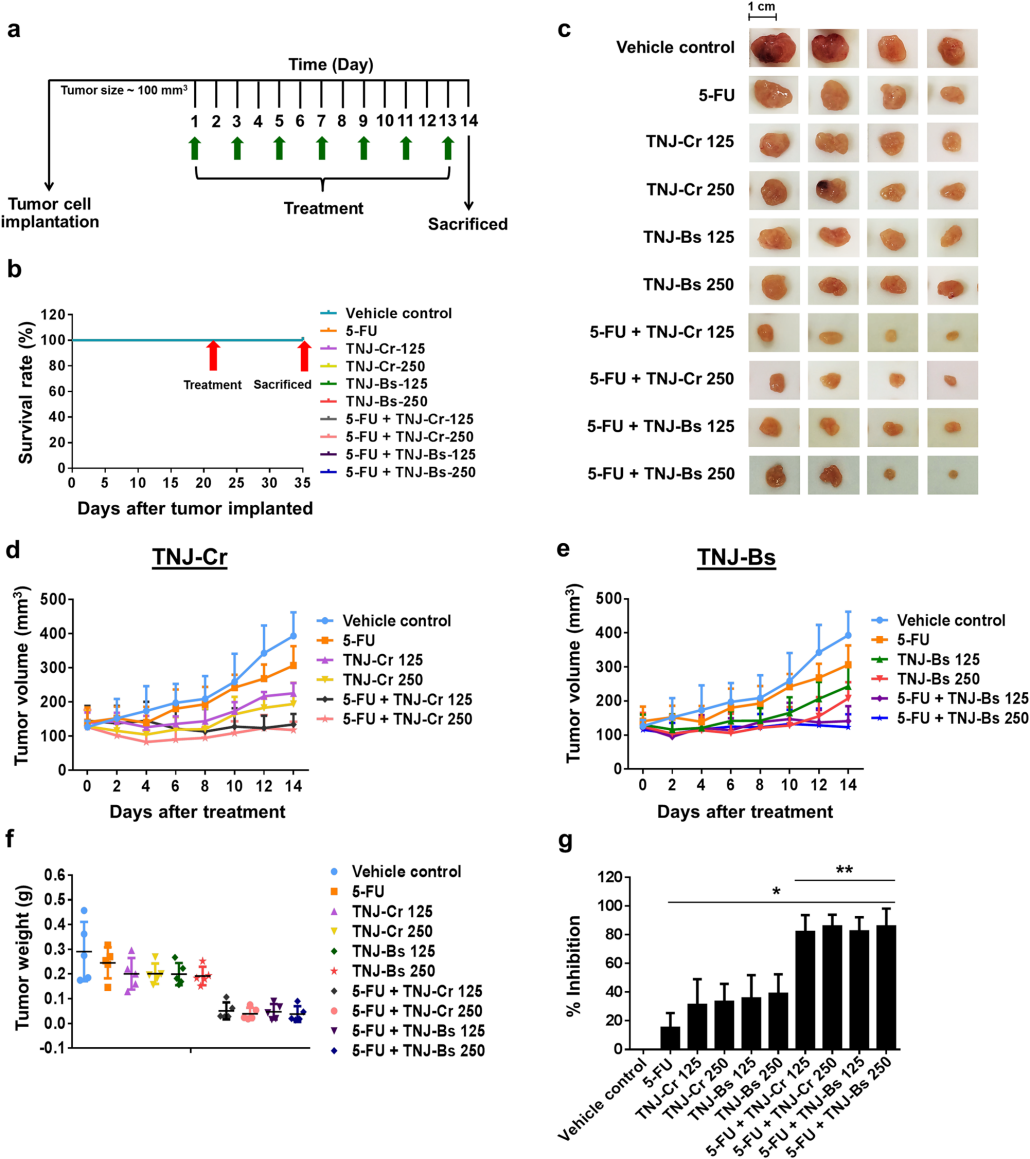
Effects of 5-FU, ethanolic extracts of TNJ-Cr and TNJ-Bs in single and combined agent treatments on KKU-100 cells-inoculated nude mice. Experimental design of the administration of 5-FU and TNJ ethanolic extracts in single or combined agent treatments (**a**). The mouse survival rates after tumor inoculation (**b**). Representative photographs of mouse tumors (**c**). Tumor volume of KKU-100 inoculated mice after treatment with 5-FU (10 mg/kg) and TNJ-Cr ethanolic extract (125 or 250 mg/kg) alone or in combination (**d**). Tumor volume of KKU-100 inoculated mice after treatment with 5-FU (10 mg/kg) and TNJ-Bs ethanolic extract (125 or 250 mg/kg) alone or in combination (**e**). Tumor weight after surgical excision (**f**). The inhibition rate of 5-FU and TNJ ethanolic extracts on tumor growth (**g**). Data are presented as mean ± SD of 5 mice in each group. *p < 0.05 indicates a significant difference between the treatments and the vehicle control group; **p < 0.05 indicates a significant difference between the single and combined agent treatments group.

**Figure 6:**
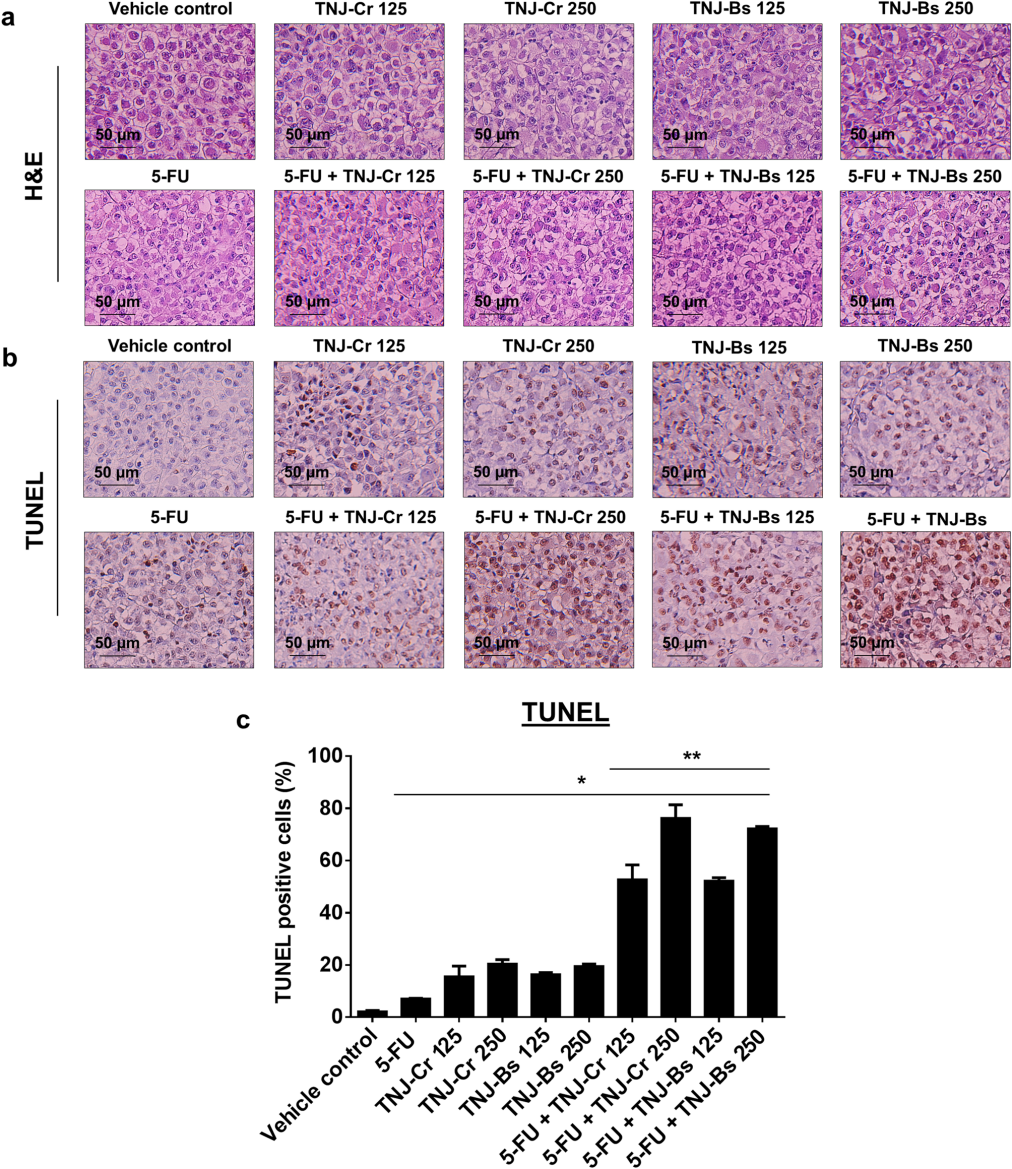
Effects of 5-FU, ethanolic extracts of TNJ-Cr or TNJ-Bs in single and combined agent treatments on tumor cells and apoptosis induction. Histopathology of the mouse tumor sections were performed by hematoxylin and eosin staining (H&E) and examined under a light microscope (**a**). Scale bar = 50 μm. In situ apoptosis of the tumor sections were detected by TUNEL staining (**b**). Scale bar = 50 μm. Bar graph shows the mean percentage of TUNEL-positive cells by counting brown-stained nucleic cells per 1000 cells in each section (**c**). *p < 0.05 indicates a significant difference between the treatments and the vehicle control group; **p < 0.05 indicates a significant difference between the single and combined agent treatments group.

After 21 days of mice tumor implantation (tumor volume reached 100 mm^3^), mice were intraperitoneal (i.p.) injected every other day with PBS as vehicle control, 5-FU, and ethanolic extracts of TNJ-Cr or TNJ-Bs alone or in the combination for 14 days (Fig. 5a). The tumor length and width of each mouse were measured by digital vernier caliper after drug treatment in each time point (Supplementary Table S1). No mice died in any group after CCA cell inoculation and treatment with the single or combined agents (Fig. 5b). Mice were sacrificed and the tumors were excised and photographed (Fig. 5c). As shown in Fig. 5d–f, mice groups treated with TNJ-Cr or TNJ-Bs ethanolic extracts alone in the dose of 125 or 250 mg/kg had a decrease in tumor volume and weights after 2 weeks of treatment when compared to the groups treated with the vehicle control and with the 5-FU alone. Moreover, the groups treated with combined 5-FU and TNJ ethanolic extracts showed a greater decrease in tumor volume. The results showed no significant difference between groups treated with the low or high doses of TNJ ethanolic extracts within either single agent or combined regimes. However, the mean tumor volume and weight in groups treated with combined 5-FU and a high dose TNJ ethanolic extracts were less than for tumors from groups treated with combined 5-FU and a low dose TNJ ethanolic extracts. Single agent treatment of 5-FU or TNJ ethanolic extracts could reduce tumor weight and growth by 15.48 ± 10.00% for 5-FU 10 mg/kg, 31.33 ± 17.75% for TNJ-Cr 125 mg/kg, 33.57 ± 12.12% for TNJ-Cr 250 mg/kg, 35.96 ± 15.89% for TNJ-Bs 125 mg/kg and 39.15 ± 13.27% for TNJ-BS 250 mg/kg when compared to the vehicle control group (Fig. 5g and Supplementary Table S2). Interestingly, the combined treatments of 5-FU and TNJ-Cr 125 or 250 mg/kg showed 82.21 ± 11.50% and 86.12 ± 7.81% tumor growth inhibition, respectively. This result was similar to combined treatments of 5-FU and TNJ-Bs 125 or 250 mg/kg which suppressed the tumor growth 82.69 ± 9.50% and 86.12 ± 12.17%, respectively. These results revealed that both TNJ-Cr and TNJ-Bs ethanolic extracts enhanced the anti-tumor activity of 5-FU with no significant difference in the nude mouse xenograft model. The hematoxylin and eosin staining and terminal deoxynucleotidyl transferase-mediated dUTP nick-end labeling (TUNEL) assay were used to further examine tumor histology and apoptosis induction in the mice (Fig. 6). Histopathology analysis of tumors in the groups treated with the single and combined agents of 5-FU and TNJ ethanolic extracts showed nuclear condensation in the tumor cells (Fig. 6a). Compared to the tumors in the vehicle control group, the TUNEL-positive cells with brown-stained nuclei were increased in groups treated with 5-FU or TNJ ethanolic extracts alone (Fig. 6b). Moreover, the tumors of groups treated with combined 5-FU and TNJ ethanolic extracts showed more intense staining of TUNEL-positive cells than the tumors of groups treated with either agent alone. Furthermore, in the groups treated with combined 5-FU and a high dose TNJ ethanolic extract (250 mg/kg) showed significantly increased TUNEL stained cells compared to groups treated with combined 5-FU and low dose TNJ ethanolic extracts (125 mg/kg) (Fig. 6c).

### In vivo toxicological evaluation

**Figure 7:**
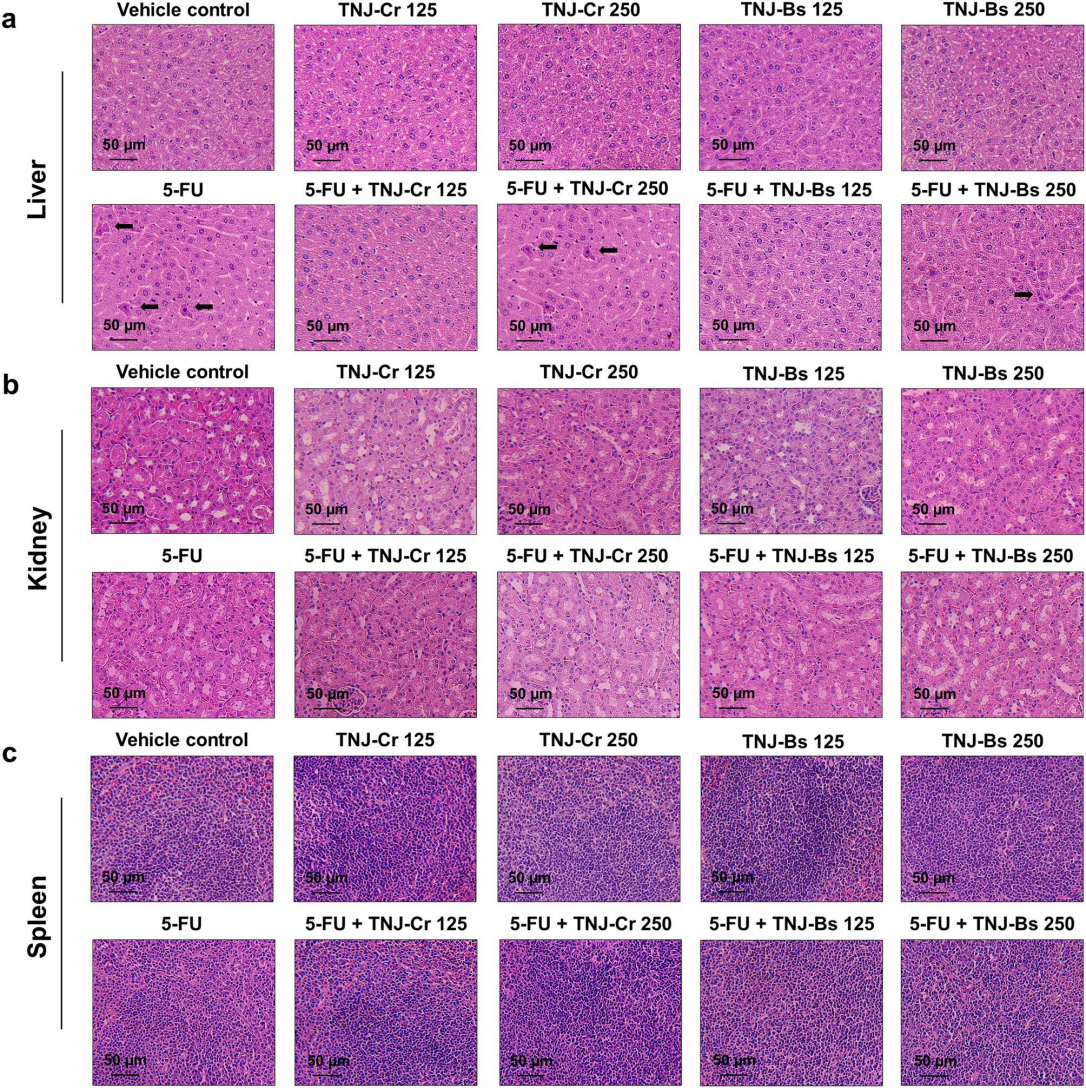
Histopathology of mice organ. After the treatment of mice with 5-FU, ethanolic extracts of TNJ-Cr or TNJ-Bs alone or in combination, the liver, kidney, and spleen of mice were excised. The tissues, liver (**a**), kidney (**b**) and spleen (**c**), were stained by hematoxylin and eosin and examined under a light microscope. The black arrow indicates hepatocyte degeneration that is often described as vacuolar or hydropic degeneration. Scale bar = 50 μm.

As shown in Table 2 and Supplementary Table S3, mean initial and final body weights of mice showed no significant changes between the vehicle control and the treatment groups. In addition, liver weights were not affected by the single agent treatment with the low or high doses of TNJ ethanolic extracts when compared to the vehicle control group. The mice with 5-FU alone or in combination with a high dose TNJ ethanolic extracts (250 mg/kg) had significantly reduced in liver weights. In contrast, the combined treatment of 5-FU and a low dose of TNJ ethanolic extracts (125 mg/kg) did not significantly change liver weights. Compared to the vehicle control group, the treatment groups with 5-FU, TNJ-Cr and TNJ-Bs ethanolic extracts alone or in combination showed no significant effects on kidney and spleen weights. Histological sections of mouse livers revealed normal hepatocyte morphology in the groups treated with the vehicle control or the single agent of TNJ-Cr or TNJ-Bs ethanolic extracts with low or high dose (Fig. 7a). Whereas the groups treated with 5-FU alone or with the combination of 5-FU and a high dose TNJ-Cr or TNJ-Bs ethanolic extracts demonstrated hepatocyte injury as shown by the vacuolar or hydropic degeneration in the hepatocytes. However, the combination treatment of 5-FU with low dose TNJ ethanolic extracts showed no hepatocellular damage (Fig. 7a). Moreover, there were no significant differences in histological findings of mouse kidney and spleen tissues between the single or combined agents treated groups and the vehicle control (Fig. 7b,c).

**Table 2:** Body weight and relative organ weight of nude mice in the vehicle control and treated groups.

Groups	Initial body weight (g)	Final body weight (g)	Organ weight (g/100 g body weight)		
			Liver	Kidney	Spleen
Vehicle control	21.57 ± 0.72	22.71 ± 1.00	8.36 ± 0.57	1.03 ± 0.11	0.62 ± 0.14
5-FU	20.72 ± 1.12	21.76 ± 0.60	7.12 ± 0.38^a^	0.98 ± 0.03	0.63 ± 0.09
TNJ-Cr 125	22.28 ± 1.04	23.16 ± 0.68	7.87 ± 0.20	1.02 ± 0.04	0.49 ± 0.05
TNJ-Cr 250	21.04 ± 1.39	22.64 ± 0.71	7.92 ± 0.38	0.95 ± 0.05	0.48 ± 0.02
TNJ-Bs 125	21.75 ± 0.42	22.74 ± 0.69	7.79 ± 0.16	0.94 ± 0.04	0.58 ± 0.02
TNJ-Bs 250	21.59 ± 1.15	22.85 ± 1.22	7.87 ± 0.23	0.93 ± 0.04	0.49 ± 0.02
5-FU + TNJ-Cr 125	20.62 ± 0.82	22.26 ± 0.52	7.77 ± 0.15	0.96 ± 0.04	0.66 ± 0.12^c^
5-FU + TNJ-Cr 250	21.91 ± 0.60	22.60 ± 0.86	6.96 ± 0.20^a^	0.99 ± 0.02	0.72 ± 0.16^d^
5-FU + TNJ-Bs 125	21.92 ± 1.36	23.09 ± 1.27	8.25 ± 0.82^b^	0.98 ± 0.08	0.64 ± 0.17
5-FU + TNJ-Bs 250	21.55 ± 0.65	22.98 ± 0.61	7.52 ± 0.23^a,f^	0.92 ± 0.01	0.66 ± 0.09^f^

Results are expressed as mean ± SD from five mice.

5-FU: 5-fluorouracil; TNJ-Cr 125: ethanolic extract of Thai noni juice Cr 125 mg/kg; TNJ-Cr 250: ethanolic extract of Thai noni juice Cr 250 mg/kg; TNJ-BS 125: ethanolic extract of Thai noni juice Bs 125 mg/kg; TNJ-Bs 250: ethanolic extract of Thai noni juice Bs 250 mg/kg.

^a^*p* < 0.05 versus vehicle control, ^b^*p* < 0.05 versus 5-FU, ^c^*p* < 0.05 versus TNJ-Cr 125, ^d^*p* < 0.05 versus TNJ-Cr 250, ^e^*p* < 0.05 versus TNJ-Bs 125, ^f^*p* < 0.05 versus TNJ-Bs 250.

## Discussion

Chemotherapy of CCA with 5-FU is often associated with adverse side effects and drug-resistance. So, the development of 5-FU combination therapy with another substance such as a natural bioactive compound from a plant or herb extract is an alternative treatment that will enhance the anticancer activity and reduce the drug resistance of 5-FU^18–20^. From our previous study, two TNJ ethanolic extracts (TNJ-Cr and TNJ-Bs) possessed anticancer activity against CCA cell lines^17^. However, the anti-CCA activities of both TNJ ethanolic extracts were not potent when used alone. In this study, for the first time we demonstrated the combined effects of 5-FU and TNJ ethanolic extracts on human KKU-100 (poorly-differentiated and drug resistant) and KKU-213B (well-differentiated) CCA cells both in vitro and in vivo. Our findings revealed that combination treatments of 5-FU and TNJ ethanolic extracts showed greater inhibition of cell viability than either single agent treatment for both KKU-100 and KKU-213B cells. However, only the combined treatment of 5-FU with TNJ-Cr ethanolic extract was toxic to the non-cancer H69 cell. It might be due to H69 cells lacking telomerase activity and being predisposed to senescence as well as consequently being more sensitive to treatment after several sub-passages^21^. In addition, both TNJ ethanolic extracts at IC_50_ concentration were not toxic to the isolated human peripheral blood mononuclear cells (PBMCs), when treated for 24 h with each TNJ ethanolic extract in vitro^17^. The type of drug interaction was determined by CI calculation and the combination treatment of 5-FU and TNJ ethanolic extracts exerted synergism at 48 h exposure in both KKU-100 and KKU-213B cells. Subdivided synergism indicated the degree of synergistic effects as a synergism (CI value range 0.30–0.70) for the combined treatment of 5-FU and TNJ-Cr or TNJ-Bs ethanolic extracts at 48 h exposure of KKU-100 cells. Using the KKU-213B cells, only 5-FU combined with TNJ-Cr ethanolic extract showed synergism, while 5-FU combined with TNJ-Bs ethanolic extract showed a moderate synergism (CI value range 0.70–0.85) at 48 h of treatment^22^. These synergistic effects permit a dose reduction for 5-FU of 5.83- and 12.86-fold in KKU-100 and KKU-213B cells, respectively, which yields a 50% inhibition in cell proliferation for a 48 h exposure time. A dose reduction of the main drug 5-FU in KKU-213B was greater than for KKU-100, which was probably due to the drug resistance properties of KKU-100 cells^23^. In addition, our results indicated a dose reduction for both TNJ-Cr and TNJ-Bs ethanolic extracts to be 3- and fourfold in KKU-100, respectively; whereas, the dose reductions of both TNJ ethanolic extracts for KKU-213B were less than twofold. This finding was correlated to the CI values of both TNJ-Cr and TNJ-Bs ethanolic extracts in the combination treatments with 5-FU of both KKU-100 and KKU-213B cells.

The mechanism underlying the combination treatment between 5-FU and TNJ ethanolic extracts in drug resistant KKU-100 was an apoptotic cell death not cell cycle arrest. Although a treatment of single agent 5-FU induced both S phase cell cycle arrest and apoptosis, the combination treatment of 5-FU and TNJ ethanolic extracts induced apoptosis by increasing the sub-G1 population of KKU-100. Our results indicated that TNJ ethanolic extracts could enhance the effect of 5-FU on drug resistant cancer cells. Cell cycle perturbation after 5-FU treatment also was found in the resistant colon (H630R10) and breast (T47DFU2.5) cancer cell lines that appeared to provide time for cancer cells to repair the damage of 5-FU^24^. Enhancement of 5-FU by herb extract was reported to overcome the acquired drug resistant cancer cell via switching the mode of action from cell cycle arrest to apoptosis^25^. Moreover, our results illustrated the combination treatment of 5-FU and TNJ ethanolic extract induced apoptosis through up-regulation of p53 and Bax. Several phenolic acids from the plant activated the expression of p53 and triggered the transcription of numerous target genes that involve apoptosis such as Bax^26,27^. These results were consistent with a previous study of noni juice from Health India Laboratories showing that the combination treatment with cisplatin induced cervical cancer cell apoptosis through p53 and Bax^15^. The up-regulation of an anti-apoptotic Bcl-2 after treating KKU-100 cells with 5-FU or TNJ ethanolic extracts alone was considered as a chemotherapeutic resistance response of the cancer cells^28^. However, 5-FU in combination with TNJ ethanolic extracts could reduce the Bcl-2 level in KKU-100 cells when compared to the treatment with 5-FU alone. Moreover, the increasing Bax/Bcl-2 ratio in the combination treatment of 5-FU and TNJ ethanolic extracts indicated increasing apoptosis in the KKU-100 cells^29^. Our findings suggest that the TNJ ethanolic extracts decrease drug resistance of 5-FU treatment with the implication that Bcl-2 is a promising target to overcome the drug resistance in CCA cells.

The experimental basis for the preclinical application of 5-FU in combination with TNJ ethanolic extracts was examined in nude mouse CCA xenograft model. Our results demonstrate that TNJ-Cr and TNJ-Bs ethanolic extracts enhance tumor suppression of 5-FU in the nude mice. Consistent with the in vitro results, apoptosis is the main effect of the combination treatment of 5-FU and TNJ ethanolic extracts on KKU-100-inoculated mice. Although no significant difference was observed in the mouse tumor volume between the 5-FU combined treatments of low (125 mg/kg) and high doses (250 mg/kg) of TNJ ethanolic extracts, the apoptotic-positive cells in the mouse tumors were increased in the combined group treated with 5-FU and high dose TNJ ethanolic extracts. This result suggests that a longer treatment time of more than 14 days may show a significant change of tumor volume between the combined treatments of 5-FU with low and high doses of TNJ ethanolic extracts. However, the average inhibition ratio of tumors in the group treated with 5-FU and a high dose TNJ ethanolic extract was greater than the group treated with 5-FU and a low dose TNJ ethanolic extract. In the in vivo toxicological evaluations, our results demonstrated that the mice treated with 5-FU and TNJ ethanolic extracts alone or in combination showed no significant adverse effects on body weights. However, the mean body weight of the mice treated with 5-FU alone was the lowest among the treatment groups. The mice liver weights were decreased in treatment groups with 5-FU alone or in the combination with a high dose of TNJ ethanolic extracts. Long term administration of 5-FU can lead to liver damage^30^ and the changing liver weight reflects the loss of liver mass due to the metabolism of 5-FU in the liver^31,32^. So, the liver damage in nude mice may be related to the side effects of 5-FU administration. However, both TNJ ethanolic extracts alone did not affect the mice liver weight. The mice treated with 5-FU and a low dose of TNJ-Cr or TNJ-Bs ethanolic extracts showed significant recovery of liver weights as well as normal hepatocytes when compared to mice treated with 5-FU alone. Therefore, hepatotoxicity in mice may be due to the 5-FU administration, and a low dose of TNJ ethanolic extracts appears to reduce the toxicity of 5-FU. Herbal medicine can reduce liver damage of 5-FU through eliminating free radicals and reduced oxidative damage from 5-FU toxicity^33^. Moreover, our findings demonstrate that TNJ ethanolic extracts alone and in combination treatment with 5-FU did not affect mouse kidney and spleen. Taken together, the TNJ ethanolic extracts enhanced the anticancer activity of 5-FU in the treatment of the drug resistant CCA cells. In addition, a low dose of TNJ ethanolic extracts appeared to reduce the toxicities of chemotherapy. However, clinical studies on the anticancer activity and the toxicity of the combination treatment of 5-FU and TNJ ethanolic extracts in the CCA patients still require further investigation.

In our previous study, the phenolic acid contents of both TNJ ethanolic extracts were partially identified based on the availability of phenolic acid standards and the fact that phenolic acids in several plant extracts possessed anticancer activity in our previous findings^34,35^. Methanolic extracts of peanut testae composed of predominantly *p*-hydroxybenzoic and *p*-coumaric acids had the antiproliferative activity against cervical, colon, leukemia and breast cancer cells^34^. Moreover, *p*-coumaric, ferulic, and sinapinic acids possessed histone deacetylase inhibitory activity against breast and cervical cancer cell lines^35^. Partial identification of phenolic acid composition in TNJ ethanolic extracts revealed that *p*-hydroxybenzoic, vanillic, and protocatechuic acids were the major phenolic acids in ethanolic extracts of TNJ^17^, which were reported to be natural anticancer agents^36–38^.

Yoshitomi et al. identified seven major compounds, asperulosidic acid, deacetylasperulosidic acid, scopoletin, morindolin, and three fatty acid glycosides, in the noni juice and they demonstrated that deacetylasperulosidic acid had an activity to reduce the blood pressure^39^. However, the anticancer activity of deacetylasperulosidic acid has not yet been reported. Some other identified compounds had been reported the anticancer activity^40–44^. Asperulosidic acid possessed the antitumorigenic effects through inhibition of AP-1 transactivation and cell transformation in the mouse epidermal JB6 cell line^40^. Scopoletin exerted the anticancer effects by triggering apoptosis, cell cycle arrest and inhibition of cell invasion^41^. Rutin reduced cell viability in the liver cancer cells^42^. Moreover, other minor active compounds in noni extracts were also reported to possess the anticancer activity such as quercetin^43^, kaempferol^43^, and chrysin^44^. The anticancer activity of TNJ ethanolic extracts is dependent on the amount and type of active compounds in the extracts, however, these compounds may be active as single or in cooperative contribution with their natural cocktails presented in the extracts. For example, dichloromethane extract of fresh noni leave showed more antiproliferative activity than the pure compounds rutin or scopoletin, suggesting that an individual compound from the plant has lost the synergistic effect of various natural ingredients^45^. Nonetheless, the existence of other promising active compounds in the two TNJ ethanolic extracts will be further investigated before the clinical trial.

In conclusion, combination treatments of 5-FU and TNJ-Cr or TNJ-Bs ethanolic extracts showed the synergistic antitumor effects on the human CCA cell lines, KKU-100 and KKU-213B. These synergistic effects can enable the dose reduction of 5-FU 5.84- and 12.86-fold for 50% cell proliferation inhibition in KKU-100 and KKU-213B cells, respectively. Apoptosis is the main mechanism for the synergism of 5-FU with TNJ ethanolic extracts on a drug resistant KKU-100 cells via the up-regulation of pro-apoptotic protein p53 and Bax. Moreover, the combined treatment of 5-FU and TNJ-Bs ethanolic extract was not toxic to the non-cancer H69 cells in vitro. Additionally, TNJ ethanolic extracts enhanced the antitumor growth of 5-FU on the mouse KKU-100 xenograft model through apoptosis induction. The treatment of 5-FU alone or in combination with high dose TNJ ethanolic extracts in KKU-100 inoculated nude mouse exerted toxic effects on mouse liver; whereas, combined treatment of 5-FU with low dose TNJ ethanolic extracts reduced the toxicity of 5-FU. Moreover, the combination treatment of 5-FU with TNJ ethanolic extracts shows no toxicity on mouse kidney and spleen. Therefore, the TNJ ethanolic extracts could be used in combination with 5-FU to reduce the toxicity and enhance the anticancer activity of the main drug. In a further study, the combination treatment of 5-FU with TNJ ethanolic extracts will be investigated in clinical trials.

## Methods

### Cell lines, culture conditions, and reagents

Two CCA cell lines, KKU-100 (poorly differentiated and drug resistant) and KKU-213B (well-differentiated) were established from Opisthorchiasis-associated Thai CCA patients, which was kindly provided by Prof. Dr. Banchob Sripa^46,47^. Both CCA cells were cultured in RPMI-1640 supplemented with 10% fetal bovine serum and antibiotics (100 U/mL penicillin and 100 μg/mL streptomycin (Gibco, USA)). Human epithelial cells line, H69 was a kind gift from Dr. D. Jefferson (Tufts University, Boston, MA, USA)^48^ and maintained in Dulbecco's Modified Eagle Medium (DMEM; Gibco, USA) supplemented with 10% FBS, 100 μg/mL streptomycin, 100 U/mL penicillin, 25 μg/mL adenine, 10 ng/mL EGF, 1 μg/mL epinephrine, 8.30 μg/mL holo-transferrin, 0.62 μg/mL hydrocortisone, 5 μg/mL insulin, and 13.60 ng/mL T3T Triiodo-l-thyronine. Cells were incubated at 37 °C in a humidified incubator with 5% CO_2_ atmosphere. 5-fluorouracil (5-FU) was purchased from Panreac Applichem (Darmstadt, Germany).

### Preparation of TNJ ethanolic extracts

Two commercial TNJ products (TNJ-Cr and TNJ-Bs) utilized in this study were purchased from the market and the same lot number was used throughout the study. Thai noni juice Chiangrai (TNJ-Cr) and Buasri (TNJ-Bs) were obtained from Chiangrainoni Co., Ltd., Thailand (https://opencorporates.com/companies/th/ 0575553000683), and Maebuasri brand of One Tumbon One Product (OTOP), Thailand (http://www.nonibuasri.com), respectively. The same lot numbers of both TNJ products were used throughout this study. TNJ products (30 mL) were stirred continuously with 70 mL of absolute ethanol at room temperature for 4 h. The extracts were filtered through No.1 Whatman filter paper and then ethanol was removed from the filtrate by rotary evaporator. The ethanolic extracts of TNJ products were lyophilized to dryness and keep at − 20 °C until used. The ethanolic extracts of TNJ-Cr and TNJ-Bs were obtained with a yield of 28.99 mg/mL and 25.69 mg/mL of the original noni juice, respectively.

### Cell viability assay

Cells were seeded in 96 well plates at a density of 8 × 10^3^ cells/well and cultured for 24 h. After that, cells were treated with solvent (0.5% ethanol + 0.5% dimethyl sulfoxide (DMSO); solvent control) and various concentrations of single agents (5-FU or ethanolic extracts of TNJ-Cr or of TNJ-Bs) for 24, 48 and 72 h. For combination treatment, cells were treated with a sub-toxic concentration of 5-FU (IC_20_) combined with various concentrations (0.06–3.00 mg/mL) of TNJ-Cr or TNJ-Bs ethanolic extracts for 24, 48 and 72 h. After incubation, the culture medium was replaced with fresh medium containing MTT and incubated at 37 °C for 2 h. DMSO was added to dissolve formazan, and the absorbance was measured at the wavelength of 550 nm by a microplate reader (EZ Read 2000, Biochrom, Cambridge, UK) using 655 nm as the reference wavelength. The cell viability was determined as a percentage by following: % cell viability = (*A*_550_ Sample − *A*_655_ Sample)/(*A*_550_ Control − *A*_655_ Control) × 100, where *A* is the absorbance.

### Determination of drug interaction

The drug interactions of 5-FU with either TNJ-Cr or TNJ-Bs ethanolic extracts were determined by calculating the CI according to the Chou-Talalay method^49^. For 50% growth inhibition, the equation of CI values is as follow: CI = (*D*_1_/*Dx*_1_) + (*D*_2_/*Dx*_2_) + *α*⋅(*D*_1_⋅*D*_2_)/(*Dx*_1_⋅*Dx*_2_), where *D*_1_ is a dose of drug 1 (5-FU) in combination with drug 2 (TNJ ethanolic extracts) to produce 50% cell viability; *Dx*_1_ is a dose of drug 1 alone to produce 50% cell viability; *D*_2_ is a dose of drug 2 in combination with drug 1 to produce 50% cell viability; *Dx*_2_ is a dose of drug 2 alone to produce 50% cell viability; *α* = 1 for mutually non-exclusive modes of drug action. CI < 1 shows a synergistic effect; CI = 1 shows an additive effect, and CI > 1 shows antagonism. The fold of dose reduction in combination treatment was indicated by DRI that provided the level of effect as compared to the dose of a single agent. The DRI was calculated using the following equation: DRI = *Dx*/*D*.

### Cell cycle analysis

To identify the mechanism under synergistic anti-CCA effects, cell cycle phase distribution was analyzed by using propidium iodide (PI) staining (Sigma Aldrich, St. Louis, MO, USA) and flow cytometry. Cells were seeded in a 5.5 cm dish plate at a density of 1 × 10^6^ cells and cultured for 24 h. Then, the cells were treated with 5-FU and TNJ ethanolic extracts alone or in combination at the synergistic concentration for 48 h exposure. After incubation, cells were harvested, washed with ice-cold phosphate-buffered saline (PBS) containing 1% glucose and fixed in 70% ethanol for 1 h on ice. The cells were washed with PBS and then incubated with RNase A (0.1 mg/mL) at 37 °C for 30 min. Cellular DNA was stained through incubation with PI (40 µg/mL) in the dark at room temperature for 45 min. DNA content was analyzed by BD FACSCanto II flow cytometer (Becton Dickinson, San Jose, CA, USA). The percentage of DNA distribution of Sub-G1, G0/G1, S and G2/M phases were determined by the BD FACSDiva software, the service was provided by Research Instrument Center, Khon Kaen University, Thailand.

### Apoptosis detection by flow cytometry

Apoptotic cells were detected by flow cytometry using double staining of Annexin V-FITC (BioLegend, San Diego, CA, USA) and PI according to the manufacturer’s instruction. Briefly, cells (1 × 10^6^) were cultured in a 5.5 cm dish plate for 24 h and then treated with 5-FU and TNJ ethanolic extracts alone or in combination at the synergistic concentration for 48 h. Cells were harvested and washed with cold PBS. The cell pellets were resuspended with Annexin V-binding buffer, and then, the cells were stained with annexin V-FITC and PI in the dark for 15 min at room temperature. Apoptotic cells were analyzed by flow cytometer and further analyzed by the BD FACSDiva software.

### Western blot analysis

Cells were cultured in a dish plate (1 × 10^6^ cells/dish) and treated with 5-FU and TNJ ethanolic extracts alone or in combination at the synergistic concentration for 48 h. Cells were harvested and lysed by RIPA buffer containing a protease inhibitor cocktail (Amresco, Ohio, USA). Total cellular proteins were determined by using Bio-Rad protein assay dye (Bio-Rad, CA, USA). An equal amount of protein was denatured in 2× loading buffer, separated by 12.5% SDS-PAGE and transferred to polyvinylidene fluoride (PVDF) membrane. The blots were blocked with 3% skim milk in PBS-T for 1 h and then incubated with primary antibody against p53 (#2524, diluted 1:1000), Bcl-2 (#2870, diluted 1:1000), Bax (#2772, diluted 1:1000), pERK1/2 (#4377, diluted 1:1000) and ERK1/2 (#9107, diluted 1:2000) (Cell Signaling, MA, USA) at 4 °C overnight. Afterward, the blots were washed with PBS-T and subsequently incubated with anti-mouse (#7076, diluted 1:2000) or anti-rabbit (#7074, diluted 1:2000) conjugated horseradish peroxidase secondary antibody (Cell Signaling, MA, USA) at room temperature for 2 h. After washing, the blots were developed with enhanced chemiluminescence (ECL) reagent (Bio-Rad, CA, USA) and immunoreactive bands were exposed to X-ray film. The relative intensity was analyzed by the ImageJ program and using total ERK1/2 as a loading control in western blot analysis.

### Anti-CCA in nude mice xenograft model

Female nude mice (BALB/CAJcl-Nu/Nu) 6–7 weeks old were purchased from Nomura Siam International, Bangkok, Thailand. The animal experiments were approved by the Institutional Animal Care and Use Committee of Khon Kaen University (approval No. IACUC-KKU-17/62; date of registration 21/03/2019), and performed according to guidelines established by the Ethical Principle and Guidelines for the Use of Animal for scientific purposes, National Research Council of Thailand. The study was carried out in compliance with the ARRIVE guidelines. Animal were housed and maintained under specific pathogen free conditions at temperature 23 ± 2 °C under a 12 h-light: 12 h-dark cycle at Northeast Laboratory Animal Center, Khon Kaen University, Khon Kaen, Thailand. After 7 days of acclimatization, 50 mice were administered subcutaneously into the right axillary with KKU-100 cells (2 × 10^6^ cells in 0.1 mL of serum-free medium mixed with Matrigel (Corning, Tewksbury, MA, USA)). Tumor volume was measured by digital vernier caliper and calculated using the following formula: tumor volume = (length × width^2^)/2. After tumor volume reached 100 mm^3^, mice were randomly divided into 10 groups (n = 5 per group; sample size calculation performed by Two independent means (Independent *t*-test)): vehicle control (received PBS), 5-FU (received 5-FU 10 mg/kg), TNJ-Cr 125 (received TNJ-Cr 125 mg/kg), TNJ-Cr 250 (received TNJ-Cr 250 mg/kg), TNJ-Bs 125 (received TNJ-Bs 125 mg/kg), TNJ-Bs 250 (received TNJ-Bs 250 mg/kg), 5-FU + TNJ-Cr 125 (received 5-FU 10 mg/kg and TNJ-Cr 125 mg/kg), 5-FU + TNJ-Cr 250 (received 5-FU 10 mg/kg and TNJ-Cr 250 mg/kg), 5-FU + TNJ-Bs 125 (received 5-FU 10 mg/kg and TNJ-Bs 125 mg/kg), 5-FU + TNJ-Bs 250 (received 5-FU 10 mg/kg and TNJ-Bs 250 mg/kg). The treatments were administrated by i.p. injection every other day for 14 days, tumor volume and body weight were also monitored. One day after the last treatment, mice were sacrificed and xenograft tumor, liver, kidney and spleen were excised and weighed. The inhibition of tumor was represented as % inhibition ratio following the equation: % Inhibition ratio = ((*A* − *B*)/*A*) × 100, where *A* was mean tumor weight of the vehicle control group and *B* was tumor weight of the treated group.

### Histopathology

The tumor, liver, kidney and spleen were fixed in 10% formalin, dehydrated, cleared in tissue processing and embedded in paraffin. The samples were cut to 4 µm thickness by microtome and placed on a glass slide. The tissue sections were deparaffinized in xylene, rehydrated in ethanol (99, 95 and 70%), and then washed with distilled water. The rehydrated tissue sections were stained with hematoxylin and eosin. Subsequently, the samples were analyzed under a light microscope.

### In situ apoptosis detection

Apoptotic cells of tumor tissue were detected by using an in situ cell death detection kit, POD (Roche Diagnostics, IN, USA) according to the manufacturer’s instructions. Tumor tissues were deparaffinized in xylene, rehydrated in ethanol (99, 95 and 70%), and then washed with distilled water. The sections were treated with proteinase K (20 µg/mL) (Roche Diagnostics, IN, USA) for 15 min at room temperature. The sections were washed with PBS and incubated with labeling enzyme, terminal deoxynucleotidyl transferase at 37 °C in a humidified chamber for 1 h. After incubation, the sections were washed with PBS and incubated with anti-fluorescein conjugated HRP antibody at 37 °C in a humidified chamber for 30 min. The sections were washed again with PBS and incubated with diaminobenzidine (DAB; Sigma Aldrich, St. Louis, MO, USA) for color development. The tissue sections were counterstained with hematoxylin and then mounted for examination under a light microscope. The TUNEL-positive cells were determined by counting brown-stained nucleic cells per 1000 cells in each section and the data was represented as the mean percentage of TUNEL-positive cells.

### Statistical analysis

Data are expressed as means ± standard deviation (SD) from three independent experiments. Statistical analysis was performed using the statistical program IBM SPSS version 19.0 for windows (SPSS Corporation, Chicago, IL, USA) and Graphpad Prism version 8. One-way analysis of variance (ANOVA) and Dunnett’s post-hoc test were used to analyze the statistical significance for multiple groups and Student’s *t*-test was used to analyze the statistical significance between two groups. The statistical significance between different groups was considered to be at *p* < 0.05.

## Supplementary Information

Supplementary Information.

## Data Availability

The datasets generated and/or analyzed during the study are available from the corresponding author on reasonable request.
