# Supplementary material for: Anticancer effects of the combined Thai noni juice ethanolic extracts and 5-fluorouracil against cholangiocarcinoma cells in vitro and in vivo
Source: Sci Rep. 2021 Jul 21;11:14866. doi: 10.1038/s41598-021-94049-z (PMC8295291; doi:10.1038/s41598-021-94049-z)
Supplement: Supplementary file 1 — Supplementary Information. [file 41598_2021_94049_MOESM1_ESM.pdf]

Supplementary information

Anticancer effects of the combined Thai noni juice ethanolic extracts and 5-fluorouracil against cholangiocarcinoma cells in vitro and in vivo

Jeerati Prompipak[1], Thanaset Senawong[1,2], Banchob Sripa[3], Albert J. Ketterman[4], Suppawit Utaiwat[1], Khanutsanan Woranam[1], Jarckrit Jeeunngoi[1] and Gulsiri Senawong[1]*

[1] Department of Biochemistry, Faculty of Science, Khon Kaen University, Khon Kaen 40002, Thailand
[2] Natural Product Research Unit, Faculty of Science, Khon Kaen University, Khon Kaen 40002, Thailand
[3] Department of Pathology, Faculty of Medicine, Khon Kaen University, Khon Kaen 40002, Thailand
[4] Institute of Molecular Biosciences, Mahidol University, Salaya Campus, Nakhon Pathom 73170, Thailand

*** Corresponding author:** E-mail: gulsiri@kku.ac.th; Tel.: +66 43009700 ext. 44478

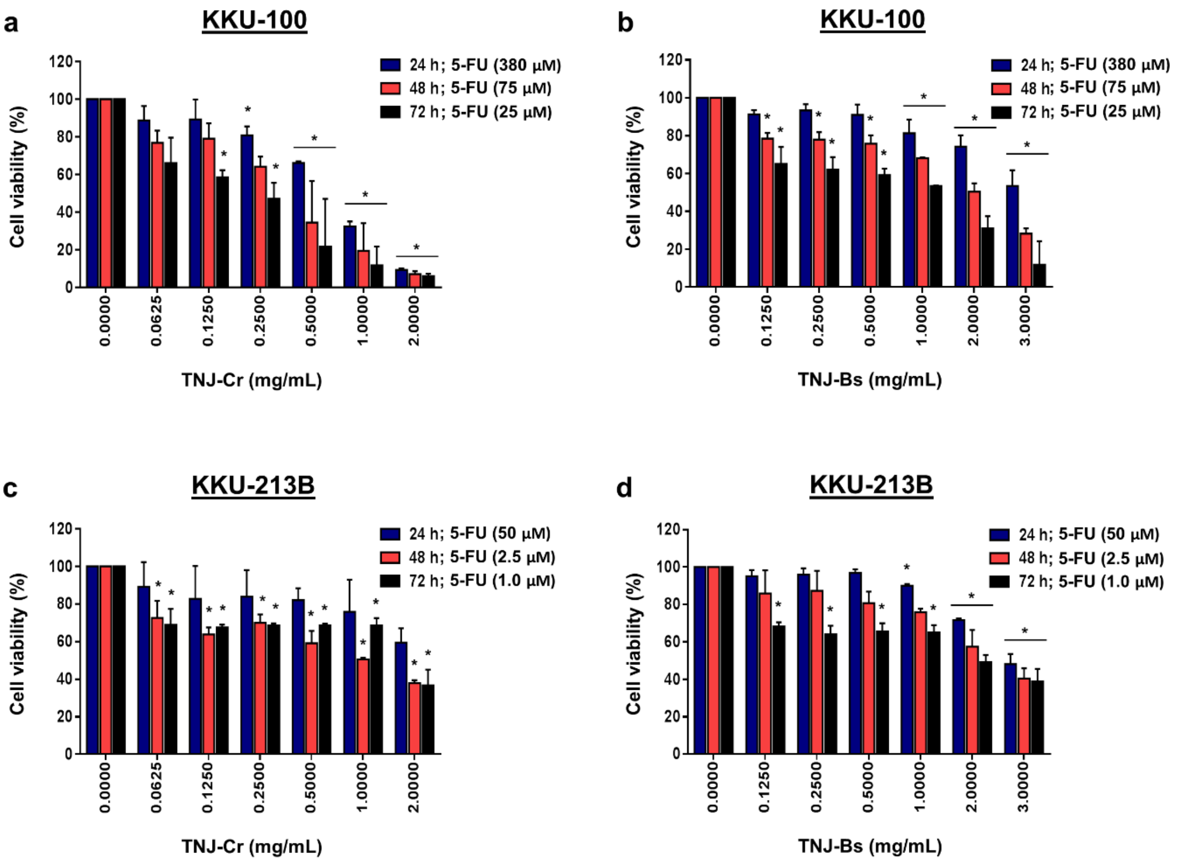

Supplementary Figure S1. The combined effects of 5-FU with ethanolic extracts of TNJ-Cr or TNJ-Bs. The sub-toxic dose of 5-FU was fixed in the combination treatment with various concentrations of each TNJ ethanolic extract on two CCA cell lines. KKU-100 cells were treated with 380, 75 and 25 µM (IC_{20}) of 5-FU for 24, 48 and 72 h, respectively (a,b). KKU-213B were treated with 50, 2.5, 1.0 µM of 5-FU for 24, 48 and 72 h, respectively (c,d). *p < 0.05 indicates significant difference between the treatments and the solvent control.

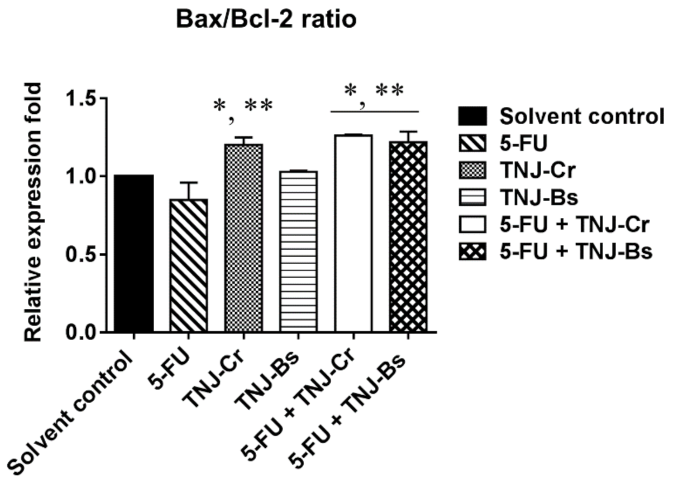

Supplementary Figure S2. The Bax/Bcl-2 ratio. Bar graph shows the mean of the Bax/Bcl-2 ratio of the relative protein expression fold. *$p < 0.05$ indicates significant difference between the treatments and the solvent control; **$p < 0.05$ indicates significant difference between other treatments and the treatment of 5-FU alone.

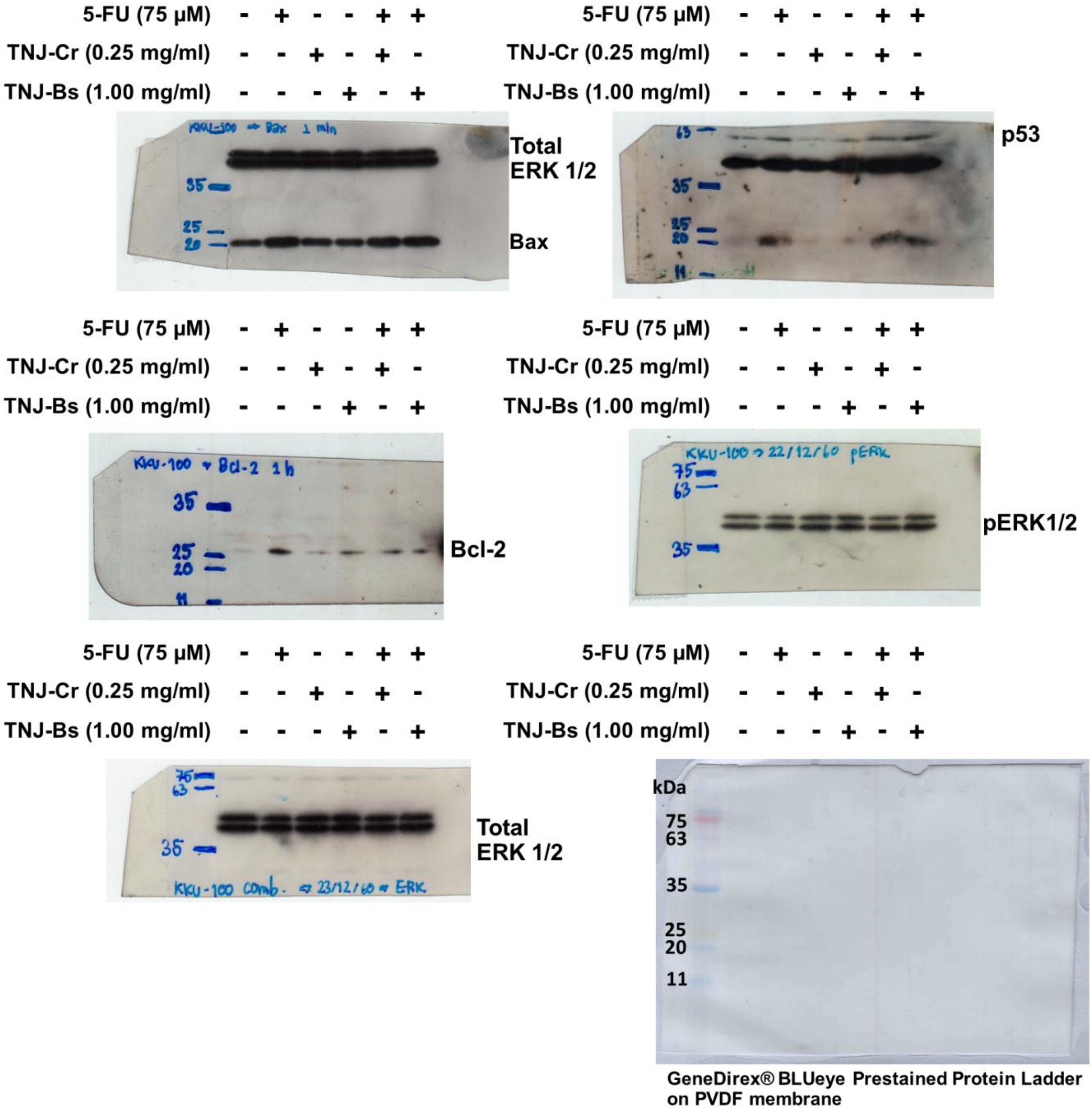

Supplementary Figure S3. The blot images represent each protein expression in western blot analysis using ECL detection reagent and exposed to X-ray film. The GeneDirex® BLUeye Prestained Protein Ladder is used for approximating the size of proteins.

Supplementary Table S1. The individual tumor measurement of each animal after the treatment in each time point. The tumor length and width were measured by digital vernier caliper in millimeters (mm) and presented as the mean from three measurements.

**Group**	**Day after treatment**	**Mouse 1**		**Mouse 2**		**Mouse 3**		**Mouse 4**		**Mouse 5**	
		**L**	**W**	**L**	**W**	**L**	**W**	**L**	**W**	**L**	**W**
**Vehicle control**	**1**	6.46	6.16	6.40	6.10	6.76	5.73	6.80	6.70	6.36	5.80
	**3**	6.73	6.40	6.26	6.20	7.80	6.40	8.03	7.80	6.20	5.60
	**5**	6.86	6.16	6.60	6.60	8.06	7.50	8.56	7.86	7.13	5.33
	**7**	7.80	6.90	7.56	6.60	7.83	7.76	8.56	8.16	6.93	5.73
	**9**	8.06	6.93	8.03	6.56	8.13	7.83	8.86	8.23	7.33	5.86
	**11**	8.93	7.66	9.96	6.76	8.53	8.43	9.66	8.66	7.93	5.90
	**13**	9.13	8.43	9.33	7.90	10.06	8.50	11.60	8.96	9.73	7.43
	**14**	9.33	9.06	9.56	8.46	9.26	9.60	10.10	9.73	9.96	8.16
**5-FU**	**1**	5.66	5.53	7.43	6.90	6.06	6.00	6.66	6.53	7.76	6.93
	**3**	6.23	5.76	7.00	6.66	6.20	5.93	7.00	6.93	8.83	7.13
	**5**	6.10	5.86	6.96	6.30	6.13	5.76	7.33	6.63	8.53	6.63
	**7**	7.96	6.73	6.96	6.96	6.10	5.90	7.83	7.33	9.66	6.96
	**9**	7.86	6.63	7.20	6.90	6.83	6.20	9.03	7.13	9.66	7.33
	**11**	8.86	7.53	7.86	7.33	8.56	6.73	9.06	7.73	10.50	7.33
	**13**	9.36	7.63	8.56	7.46	9.26	6.63	9.63	7.96	11.36	7.46
	**14**	9.96	7.63	9.83	7.33	9.76	6.96	10.56	8.13	11.53	8.23
**TNJ-Cr 125**	**1**	7.06	6.33	6.16	5.56	6.90	6.56	7.10	7.10	6.53	6.26
	**3**	7.90	6.70	6.43	6.33	6.46	6.10	7.13	6.90	6.96	6.63
	**5**	7.03	6.80	6.20	5.50	6.30	6.16	7.03	7.00	6.66	5.96
	**7**	6.96	6.96	6.36	6.00	6.60	6.23	7.10	6.70	7.26	6.20
	**9**	6.76	6.50	6.10	5.56	6.60	6.36	7.73	7.50	6.40	6.33
	**11**	7.13	6.66	7.46	6.10	7.66	6.90	7.66	7.63	7.00	6.80
	**13**	7.96	7.63	7.56	6.96	7.93	7.26	8.23	7.90	8.83	6.70
	**14**	7.83	7.63	7.76	6.93	7.96	7.33	9.16	8.13	8.23	6.90

Supplementary Table S1 continued.

Group	Day after treatment	Tumor measurement (mm)									
		Mouse 1		Mouse 2		Mouse 3		Mouse 4		Mouse 5	
		L	W	L	W	L	W	L	W	L	W
TNJ-Cr 250	1	6.96	6.63	5.70	5.66	6.06	5.16	6.50	6.23	8.03	6.90
	3	6.56	5.93	5.90	5.80	6.33	5.13	5.96	5.66	7.90	6.80
	5	6.50	5.66	5.90	5.26	6.56	5.30	5.33	5.13	7.06	7.00
	7	7.03	5.90	6.40	5.86	5.90	5.13	5.83	5.23	7.63	7.20
	9	7.03	5.90	6.63	6.33	5.96	5.16	6.90	4.80	7.90	7.60
	11	8.96	6.33	7.36	6.70	6.83	5.90	7.86	5.23	8.76	7.50
	13	9.03	6.63	7.33	6.90	7.73	6.10	7.76	5.96	9.46	7.40
	14	8.53	6.80	7.43	7.03	7.66	6.26	7.76	6.10	9.83	7.73
TNJ-Bs 125	1	5.96	5.93	6.16	6.13	6.33	6.30	7.60	6.90	6.36	6.23
	3	5.26	5.13	6.30	6.10	6.56	5.23	8.46	6.80	6.06	5.96
	5	5.50	5.26	6.63	5.90	6.46	5.33	8.56	7.00	6.06	6.03
	7	6.26	5.46	7.36	6.36	6.93	5.70	8.46	7.10	6.86	6.33
	9	6.43	5.46	7.06	6.53	6.90	5.63	8.66	7.03	6.83	6.40
	11	6.60	5.96	7.36	6.70	7.63	5.80	8.83	7.33	7.63	6.80
	13	7.10	6.96	8.13	7.20	7.80	6.90	8.83	7.63	8.06	7.13
	14	8.36	6.90	9.03	7.66	8.06	7.13	9.16	8.16	9.06	7.26
TNJ-Bs 250	1	7.06	6.86	6.66	6.20	5.73	5.56	5.83	5.56	6.96	6.60
	3	6.56	6.13	6.16	5.86	6.07	5.73	5.36	5.36	6.20	6.03
	5	7.26	6.30	6.03	5.96	6.36	5.90	5.50	5.46	6.36	6.30
	7	7.23	6.33	6.53	5.16	6.46	6.00	5.83	5.20	5.93	5.83
	9	7.13	6.73	5.83	5.80	6.90	6.50	5.80	5.23	6.36	6.20
	11	7.66	7.00	6.26	5.70	6.86	6.43	5.66	5.20	6.60	6.33
	13	7.86	7.56	6.96	6.10	7.23	6.80	6.23	5.96	6.96	6.43
	14	8.43	8.33	7.83	6.56	8.06	7.70	7.76	6.53	7.93	6.60

Supplementary Table S1 continued.

Group	Day after treatment	Tumor measurement (mm)									
		Mouse 1		Mouse 2		Mouse 3		Mouse 4		Mouse 5	
		L	W	L	W	L	W	L	W	L	W
5-FU + TNJ-Cr 125	1	6.80	6.26	6.73	5.80	7.20	6.23	7.76	7.10	6.86	6.43
	3	6.40	6.10	6.76	5.76	7.03	6.13	7.36	6.83	6.93	6.60
	5	6.40	6.33	6.83	5.86	7.13	5.96	7.40	7.23	6.96	6.73
	7	6.03	5.56	6.96	5.53	6.56	5.86	7.93	6.70	6.33	6.10
	9	6.20	5.73	6.66	5.20	6.20	5.66	7.96	6.46	6.16	5.90
	11	6.53	6.16	5.70	5.66	6.36	5.83	7.96	6.66	6.86	6.33
	13	6.50	5.73	6.36	5.80	6.43	6.13	7.56	6.43	6.63	6.13
	14	6.93	5.66	7.20	6.53	6.13	5.93	7.56	7.06	6.00	6.00
5-FU + TNJ-Cr 250	1	5.40	4.70	6.46	6.43	6.90	6.76	6.20	5.50	7.76	6.93
	3	4.93	4.60	6.33	6.20	6.50	6.36	5.63	5.06	6.83	6.13
	5	4.60	3.96	5.53	5.23	6.50	6.36	5.70	5.50	5.93	5.30
	7	5.53	4.70	6.33	6.16	6.46	6.30	5.26	5.20	5.90	4.76
	9	5.63	4.66	6.30	6.10	6.43	6.40	5.36	5.16	5.86	5.60
	11	5.93	4.66	7.20	6.10	7.46	6.20	6.16	5.16	6.83	5.93
	13	5.90	4.83	7.46	6.50	7.73	6.23	6.26	5.60	6.93	6.36
	14	6.00	5.70	6.80	6.40	7.36	6.23	6.13	5.40	6.76	5.93
5-FU + TNJ-Bs 125	1	6.40	5.96	6.70	6.43	6.80	5.76	6.70	6.36	6.16	6.03
	3	5.60	4.76	6.36	5.73	6.33	5.60	6.43	5.90	6.13	5.56
	5	6.06	5.60	8.00	6.63	6.26	5.93	6.30	6.20	6.23	5.80
	7	6.06	5.66	6.96	6.93	6.20	5.43	6.46	6.10	5.83	5.66
	9	6.10	5.70	7.40	6.76	7.50	6.66	6.76	6.43	6.23	6.10
	11	5.70	5.56	7.23	6.80	7.53	6.90	7.10	6.76	6.56	6.46
	13	6.36	5.73	7.03	6.90	7.30	6.20	6.86	6.80	6.23	6.06
	14	6.53	6.30	7.20	7.00	7.00	6.00	7.26	7.13	6.16	5.33

Supplementary Table S1 continued.

Group	Day after treatment	Tumor measurement (mm)									
		Mouse 1		Mouse 2		Mouse 3		Mouse 4		Mouse 5	
		**L**	**W**	**L**	**W**	**L**	**W**	**L**	**W**	**L**	**W**
**5-FU + TNJ-Bs 250**	**1**	5.73	5.60	6.36	6.23	6.50	6.30	6.60	5.93	6.96	5.86
	**3**	5.13	5.00	6.43	6.23	6.03	5.56	5.96	5.46	7.06	6.43
	**5**	5.20	4.90	6.43	6.43	6.73	6.13	6.23	6.13	6.93	6.40
	**7**	5.16	4.86	6.90	6.90	6.23	6.16	6.23	5.80	7.43	6.86
	**9**	5.56	5.16	6.86	6.66	7.43	6.50	5.70	5.53	6.93	6.43
	**11**	5.73	5.23	6.93	6.83	7.10	6.66	6.10	5.66	7.20	6.80
	**13**	6.23	5.83	6.46	6.43	6.80	6.03	6.20	5.73	7.23	7.00
	**14**	6.33	6.10	6.26	5.96	6.40	5.80	6.40	6.16	6.86	6.76

5-FU: 5-fluorouracil; TNJ-Cr 125: ethanolic extract of Thai noni juice Cr 125 mg/kg; TNJ-Cr 250: ethanolic extract of Thai noni juice Cr 250 mg/kg; TNJ-BS 125: ethanolic extract of Thai noni juice Bs 125 mg/kg; TNJ-Bs 250: ethanolic extract of Thai noni juice Bs 250 mg/kg; L: tumor length; W: tumor width.

Supplementary Table S2. The individual mouse tumor weight after surgical excision.

**Group**	**Tumor weight (g)**				
	**Mouse 1**	**Mouse 2**	**Mouse 3**	**Mouse 4**	**Mouse 5**
**Vehicle control**	0.457	0.362	0.275	0.185	0.175
**5-FU**	0.146	0.254	0.239	0.317	0.270
**TNJ-Cr 125**	0.220	0.200	0.160	0.129	0.296
**TNJ-Cr 250**	0.201	0.157	0.183	0.269	0.196
**TNJ-Bs 125**	0.223	0.169	0.267	0.182	0.157
**TNJ-Bs 250**	0.194	0.151	0.252	0.180	0.183
**5-FU + TNJ-Cr 125**	0.030	0.027	0.028	0.107	0.062
**5-FU + TNJ-Cr 250**	0.019	0.075	0.054	0.024	0.023
**5-FU + TNJ-Bs 125**	0.090	0.020	0.067	0.043	0.016
**5-FU + TNJ-Bs 250**	0.020	0.014	0.018	0.047	0.090

5-FU: 5-fluorouracil; TNJ-Cr 125: ethanolic extract of Thai noni juice Cr 125 mg/kg; TNJ-Cr 250: ethanolic extract of Thai noni juice Cr 250 mg/kg; TNJ-BS 125: ethanolic extract of Thai noni juice Bs 125 mg/kg; TNJ-Bs 250: ethanolic extract of Thai noni juice Bs 250 mg/kg.

Supplementary Table S3. The individual mouse body weight at each time point.

Group	Day after treatment	Body weight (g)				
		Mouse 1	Mouse 2	Mouse 3	Mouse 4	Mouse 5
Vehicle control	1	21.82	20.81	21.12	21.41	22.69
	3	22.16	20.71	21.40	21.94	23.36
	5	22.45	21.08	21.85	22.46	23.55
	7	22.16	20.72	21.62	22.00	23.02
	9	22.44	21.01	22.04	22.15	23.79
	11	23.13	21.61	21.85	22.51	23.78
	13	22.65	21.59	21.00	22.45	23.20
	14	23.28	21.75	21.49	23.33	23.67
5-FU	1	22.08	19.45	21.7.0	20.00	20.40
	3	21.36	19.38	20.94	20.04	20.73
	5	21.71	20.26	21.38	20.34	21.46
	7	21.08	19.65	21.12	19.55	20.97
	9	21.42	20.58	21.86	20.85	22.24
	11	21.97	20.56	22.47	20.60	22.20
	13	22.09	20.61	21.97	20.91	22.74
	14	22.56	21.04	21.94	21.29	22.01
TNJ-Cr 125	1	23.31	22.14	21.44	21.13	23.40
	3	24.13	22.45	22.12	21.75	23.57
	5	23.63	22.57	22.20	21.89	23.70
	7	23.22	22.33	21.89	21.44	23.61
	9	23.49	22.20	22.37	22.33	23.69
	11	24.04	22.62	22.92	22.86	24.64
	13	23.24	22.05	21.88	22.09	23.62
	14	23.77	22.78	22.51	22.70	24.02

Supplementary Table S3 continued.

Group	Day after treatment	Body weight (g)				
		Mouse 1	Mouse 2	Mouse 3	Mouse 4	Mouse 5
TNJ-Cr 250	1	22.94	21.96	19.40	20.42	20.50
	3	22.41	21.61	20.69	20.08	20.39
	5	22.49	21.62	21.54	20.53	21.20
	7	22.10	20.89	20.62	20.31	20.50
	9	22.89	21.59	21.56	21.14	21.45
	11	22.69	21.81	22.06	21.70	21.56
	13	22.59	21.34	21.51	21.48	21.11
	14	23.72	22.92	22.52	22.04	21.99
TNJ-Bs 125	1	21.31	21.88	21.53	22.41	21.59
	3	20.65	21.54	22.13	22.42	21.22
	5	21.18	21.66	22.24	23.80	22.03
	7	21.30	21.41	21.81	23.67	21.99
	9	21.86	21.74	22.24	24.00	21.94
	11	22.45	21.98	23.00	23.75	22.75
	13	21.37	21.86	22.28	22.95	21.66
	14	21.93	22.26	22.97	23.74	22.80
TNJ-Bs 250	1	23.00	21.52	22.50	20.42	20.50
	3	22.29	20.65	21.95	20.13	23.46
	5	23.68	22.21	23.44	21.17	24.73
	7	23.17	22.31	23.32	21.10	25.31
	9	23.15	22.17	23.52	20.82	24.09
	11	23.49	22.85	23.24	21.04	24.91
	13	22.31	22.03	22.46	20.57	24.42
	14	22.75	22.39	23.21	21.27	24.63

Supplementary Table S3 continued.

Group	Day after treatment	Body weight (g)				
		Mouse 1	Mouse 2	Mouse 3	Mouse 4	Mouse 5
5-FU + TNJ-Cr 125	1	19.74	21.67	20.54	21.23	19.93
	3	20.26	21.03	20.17	21.27	19.86
	5	20.94	22.04	20.98	22.01	20.41
	7	20.55	21.78	20.58	22.18	20.54
	9	20.98	21.61	21.24	22.45	20.78
	11	21.01	22.82	21.35	23.01	21.48
	13	22.25	23.05	22.02	23.06	22.65
	14	21.70	22.54	21.75	22.90	22.41
5-FU + TNJ-Cr 250	1	21.36	22.81	21.34	22.02	22.04
	3	21.68	23.17	20.64	21.09	21.37
	5	22.04	23.23	21.39	21.47	22.01
	7	21.30	23.17	20.80	21.24	21.94
	9	21.76	23.90	21.34	21.57	22.06
	11	22.29	23.90	21.41	21.96	22.68
	13	22.39	24.81	22.19	22.49	23.40
	14	22.47	23.95	21.88	21.83	22.87
5-FU + TNJ-Bs 125	1	20.97	20.57	22.44	24.01	21.62
	3	21.32	20.56	23.25	23.57	21.66
	5	21.75	20.81	23.20	24.13	22.48
	7	22.13	21.14	23.54	24.26	22.09
	9	22.25	21.16	23.37	24.09	22.46
	11	21.88	20.98	23.85	24.12	22.69
	13	22.44	22.01	24.46	24.08	22.65
	14	22.42	21.39	24.45	24.21	22.98

Supplementary Table S3 continued.

Group	Day after treatment	Body weight (g)				
		**Mouse 1**	**Mouse 2**	**Mouse 3**	**Mouse 4**	**Mouse 5**
**5-FU + TNJ-Bs 250**	**1**	20.97	21.66	22.54	21.63	20.95
	**3**	21.71	21.16	22.47	21.36	20.93
	**5**	22.03	21.77	23.02	21.65	21.31
	**7**	22.35	21.65	23.02	21.74	21.04
	**9**	22.46	22.16	23.11	22.01	20.90
	**11**	22.34	22.37	23.28	21.79	21.39
	**13**	23.09	22.70	23.68	22.60	22.12
	**14**	23.08	22.63	23.92	23.01	22.28

5-FU: 5-fluorouracil; TNJ-Cr 125: ethanolic extract of Thai noni juice Cr 125 mg/kg; TNJ-Cr 250: ethanolic extract of Thai noni juice Cr 250 mg/kg; TNJ-BS 125: ethanolic extract of Thai noni juice Bs 125 mg/kg; TNJ-Bs 250: ethanolic extract of Thai noni juice Bs 250 mg/kg.
